# Genome-wide analysis of the U-box E3 ubiquitin ligase enzyme gene family in tomato

**DOI:** 10.1038/s41598-020-66553-1

**Published:** 2020-06-12

**Authors:** Bhaskar Sharma, Joemar Taganna

**Affiliations:** 1 TERI School of Advanced Studies, 10 Institutional Area, Vasant Kunj, New Delhi, Delhi 110070 India; 2 School of Life and Environmental Sciences, Faculty of Science, Engineering, and Built Environment, Deakin University, Geelong, VIC-3220 Australia; 3 SciBiz Informatics, 2/F Unit 3 CFI Building, Maharlika Highway, Brgy. Guindapunan, Palo, Leyte 6501 Philippines

**Keywords:** Plant stress responses, Gene ontology, Phylogeny, Genome duplication

## Abstract

E3 ubiquitin ligases are a central modifier of plant signaling pathways that act through targeting proteins to the degradation pathway. U-box E3 ubiquitin ligases are a distinct class of E3 ligases that utilize intramolecular interactions for its scaffold stabilization. U-box E3 ubiquitin ligases are prevalent in plants in comparison to animals. However, the evolutionary aspects, genetic organizations, and functional fate of the U-box E3 gene family in plant development, especially in tomato is not well understood. In the present study, we have performed *in-silico* genome-wide analysis of the U-box E3 ubiquitin ligase gene family in *Solanum lycopersicum*. We have identified 62 U-box genes with U-box/Ub Fusion Degradation 2 (UFD2) domain. The chromosomal localization, phylogenetic analysis, gene structure, motifs, gene duplication, syntenic regions, promoter, physicochemical properties, and ontology were investigated. The U-box gene family showed significant conservation of the U-box domain throughout the gene family. Duplicated genes discerned noticeable functional transitions among duplicated genes. The gene expression profiles of U-box E3 family members show involvement in abiotic and biotic stress signaling as well as hormonal pathways. We found remarkable participation of the U-box gene family in the vegetative and reproductive tissue development. It is predicted to be actively regulating flowering time and endosperm formation. Our study provides a comprehensive picture of distribution, structural features, promoter elements, evolutionary relationship, and gene expression of the U-box gene family in the tomato. We predict the crucial participation of the U-box gene family in tomato plant development and stress responses.

## Introduction

Ubiquitin mediated proteasomal degradation is one of the major mechanisms for post-translational regulation of gene expression and protein quality control in eukaryotes^1,2^. The Ubiquitin Proteasome System (UPS) degrades the aberrant or truncated, active, and short-lived proteins from various cellular pathways and thereby controls the protein loads of the cell^3–5^. The major characteristic feature of the ubiquitination is the addition of the ubiquitin protein molecule (*M*_*r*_: 8.5 kDa) on the lysine residues of the acceptor protein^4–7^. The ubiquitination is mediated by a three-step enzymatic process. Ubiquitin forms a thioester bond in an ATP-dependent reaction at cysteine residue with ubiquitin-activating enzyme E1. It is followed by the transfer of the ubiquitin to ubiquitin-conjugating enzyme E2, at conserved cysteine residue on the active site. Then, the ubiquitin ligase enzyme E3 mediates the transfer of ubiquitin to the target protein. An isopeptide bond is formed between the carboxyl terminus of ubiquitin and the ε-amino group of a lysine residue on the target protein. The process utilizes the 26S proteasome (made up of 19S regulatory particles and 20S core proteases) for the degradation of the ubiquitinated proteins^3–5,8^. The E3 ubiquitin ligases are the largest family among all three enzymes and classified into different families based on their structure, function, and substrate specificity. The major classes of the E3 ubiquitin ligases are; RING (Really Interesting New Gene), HECT (Homologous to E6-associated protein C terminus), CRL (Cullin-RING ligase) and U-box^5^. They mediate the transfer of the ubiquitin-protein to the substrate by either generating an intermediate complex (E3 and Ubiquitin) or directly^9–11^.

The U-box E3 ubiquitin ligases are family of proteins with a U-box motif containing 70 amino acids^12,13^. The structure of the U-box is similar to RING-type E3 ligases but lacks the zinc chelating residues, held by hydrogen bonds and salt bridges without ionic association^12,14,15^. The U-box domain misses the feature of the RING-finger domain which utilizes cysteine and histidine to chelate the zinc. It utilizes the intramolecular interactions, especially hydrogen bonds to stabilize the U-box scaffold^16,17^. The U-box domain was originally discovered as Ub Fusion Degradation 2 (UFD2) protein in yeast^17,18^. The U-box E3 enzymes add a small fraction when compared to other plant E3 enzymes but are far more in number when compared with *Saccharomyces cerevisiae*^19^. Previously identified U-box gene family members are 66 in *Arabidopsis thaliana*^20^, 101 in *Brassica rapa*^21^, 30 in *Chlamydomonas reinhardtii*^22^, 125 in *Glycine* max^23^, 21 in *Homo sapiens*, 77 in *Oryza sativa*^24^, 2 in *Saccharomyces cerevisiae*^19^ and 56 in *Vitis vinifera*^25^. The variety in the number of U-box genes in plants is linked with the genome evolution and duplication. It contributes to the expansion of the gene family that ultimately leads to the diverse biological functions in the organisms^26^. The U-box proteins are also featured with classical protein-protein interaction motifs such as armadillo repeat region, elongation factor 3 (EF3), protein phosphatase 2A (PP2A) and yeast kinase TOR1 repeats in addition to the U-box domain and predicted to be involved in the various plant developmental processes and stress signaling^26,27^. U-box proteins were found to interfere with abscisic acid responses in *A. thaliana* (At). AtPUB9, 18, 19, and PUB44 were identified to interrupt ABA biosynthesis directly or through signal transduction. AtPUB9 regulates the transcription factor ABI3 and causes increased ABA sensitivity during seedling germination^28^. Likewise, AtPUB18 and 19 also induce ABA hypersensitivity and thus, negatively regulate the ABA^29,30^. AtPUB44 ubiquitinates the AAO3 (abscisic aldehyde oxidase 3) via 26 proteasome and affects the ABA biosynthesis^31^. The abiotic and biotic stress up-regulates the expression of U-box genes in the Arabidopsis and Nicotiana^32^. The plant U-box proteins were also found to be involved in self-incompatibility in *Brassica rapa* where ARC1, a U-box E3 ligase, during rejection of self-incompatible pollen, ubiquitinates and degrades the S-receptor kinase^33^. Therefore, the U-box gene family is an important E3 ubiquitin ligase that influences many plants signaling pathways and acts differently than other E3 enzyme classes. The evolution of the U-box gene family in the tomato is largely unknown. Tomato has many unique features such as sympodial shoots, compound leaves, and fleshy fruits that makes it an interesting model plant for study than other model plants such as Rice and Arabidopsis^34^. Tomato cultivation is challenged by pathogen attacks and adverse environmental factors which make a significant loss every year^35–37^. Therefore, the identification and characterization of the vital mechanisms involved in the various stress and developmental pathways have become mandatory to deal with the challenges. Emerging evidences reveal that the ubiquitin proteasomal degradation system participates in biotic, abiotic, hormonal and various plant developmental pathways^38–40^. The identification of the U-box gene family members in the tomato will help to understand the evolutionary and functional aspects of ubiquitin proteasomal degradation system in plants and the extent of involvement of the U-box E3 ubiquitin ligase in the cellular systems and signaling pathways. It will ultimately support to develop efficient approaches for agricultural challenges and aid in our understanding on the role of ubiquitin proteasomal degradation in plant system.

## Results

### Identification and characterization of U-box gene family

The HMM (Hidden Markov Model) profile of the U-box domain was obtained from the Pfam database. The HMMER tools convert the multiple sequence alignment into the position-specific scoring system, therefore used frequently for large scale sequence analysis^41^. We obtained 62 putative sequences using HMMER with default parameters and a significant e-value of 0.01 against the *Solanum lycopersicum* genome sequence database (Taxonomy ID: 4081) excluding redundant sequences. We have analyzed the molecular weight, locus ID, chromosomal location, number of amino acids, gene length, iso-electric point, instability index, grand average of hydropathy, introns, class, nomenclature, and subcellular localization of all the U-box genes (Supplementary Table S1). The molecular weight for the U-box gene family ranges from 20237.43 to 165856.04 and the number of amino acids ranges from 179 to 1482. The pI range was 4.95 to 9.12. Most of the proteins were predicted to be unstable and hydrophilic. SlU-box 21 was predicted to occupy maximum and SlU-box 19 with a minimum volume of aliphatic side chains.

### Gene structure and motif analysis

**Figure 1:**
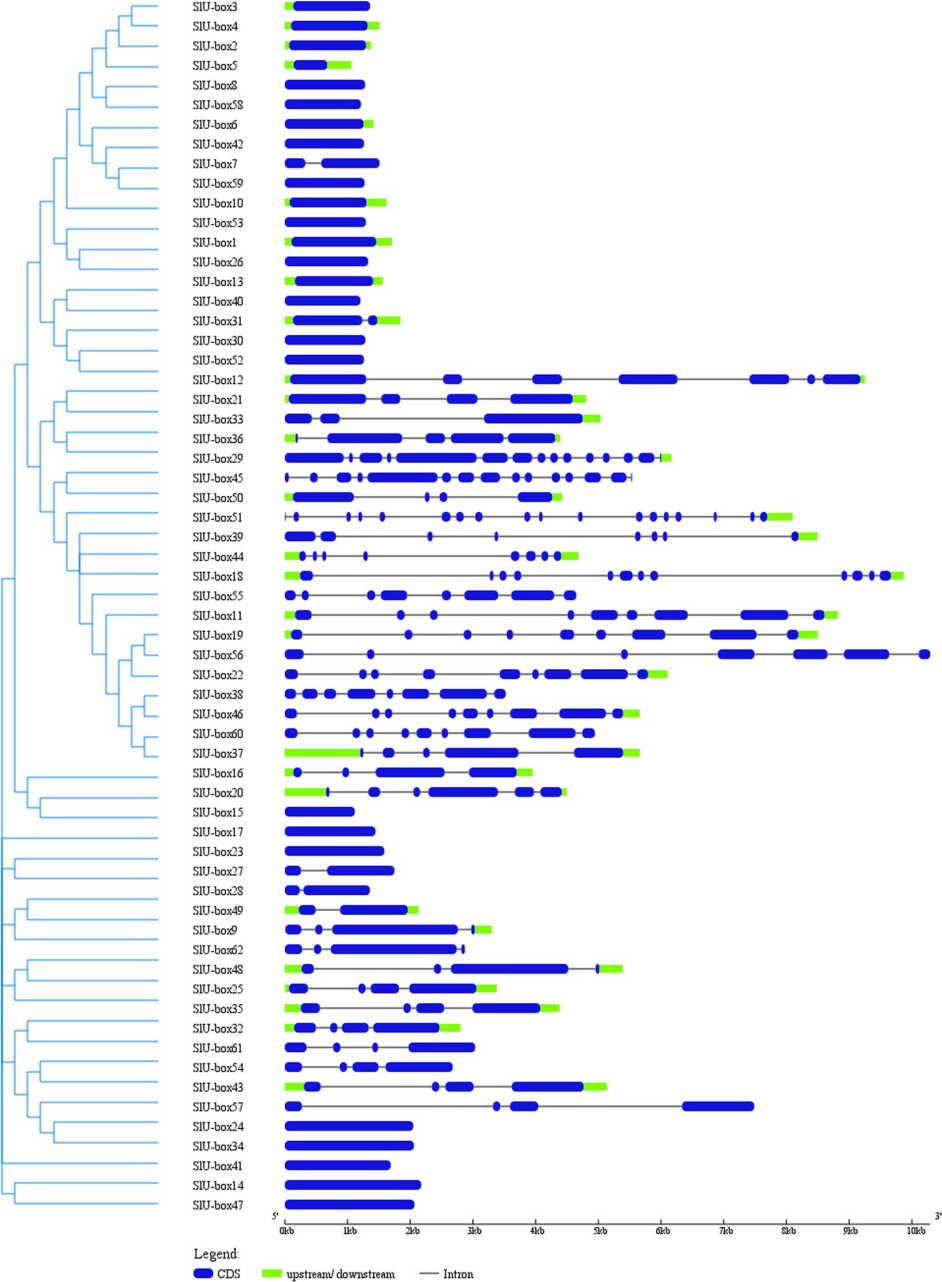
The exon/intron distribution of the 62 tomato U-box gene family was analyzed by the GSDS tool. The coding sequences were compared with the corresponding genomic sequences. The blue box represents the CDS; the continuous black line represents the intron region and green boxes represent upstream/downstream regions.

We identified and analyzed the structural organization of the U-box gene family where the number of exons varied from one to eighteen (Fig. 1). Around 66% of the Class 1 genes had no introns while sharing almost similar exon length, suggesting genetic conservation. The maximum number of introns was found in Class 3 and 4 members with significant structural differences. The 40% of all tomato U-box members were characterized by only one exon. It is evidence of functional conservation among U-box gene family members. Our results predict the ubiquitin ligase activity in all identified U-box gene family in tomato. The structural organization also illustrate the diversity among the U-box gene family members. The number of exons states the acquired assorted functional capabilities of the genes. The acquisition of numerous exons and introns pattern could be a major consequence of the U-box gene family expansion in tomato.

**Figure 2:**
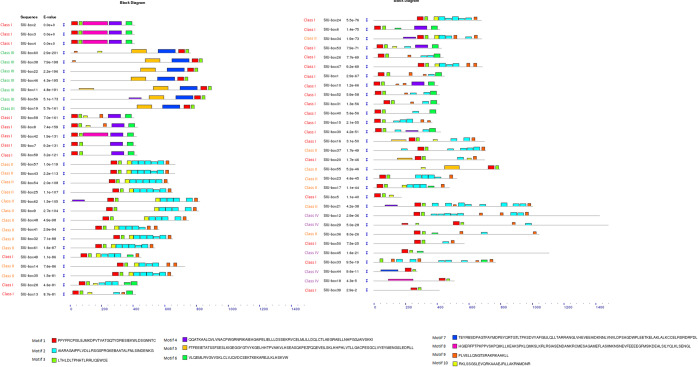
Using MEME suite 10 motifs were discovered in a total of 62 tomato U-box members. Their sequence and organization are represented by different colors.

All identified U-box genes were analyzed for the presence of the novel and ungapped motifs using MEME suite utilizing a two-component finite mixture model (Fig. 2). A total of ten motifs were detected and distributed among all the U-box members (Supplementary Table 2). Motif 1 and 3 were present throughout the tomato U-box gene family. Motif 2 was prevalent exclusively, in Class 2 and a few members of other classes. Motif 4 and 8 were mostly present in Class 1 while motif 5 was characteristic of Class 3 members which may serve a distinct biological function. The symmetric and positional features of the identified motifs propound not only the reservation of U-box domain functional facets but also the gathering of additional new domains over the course of evolution. The discovery of the ten novel motifs throughout the U-box gene family provides evidence for sharing biological functions. The common motifs patterns among the sequences are indicative of conserved evolutionary relatedness and similar cellular functions^42^. Therefore, it can be inferred that all the genes are involved in the ubiquitin ligation.

### Phylogenetic analysis and chromosomal localization

**Figure 3:**
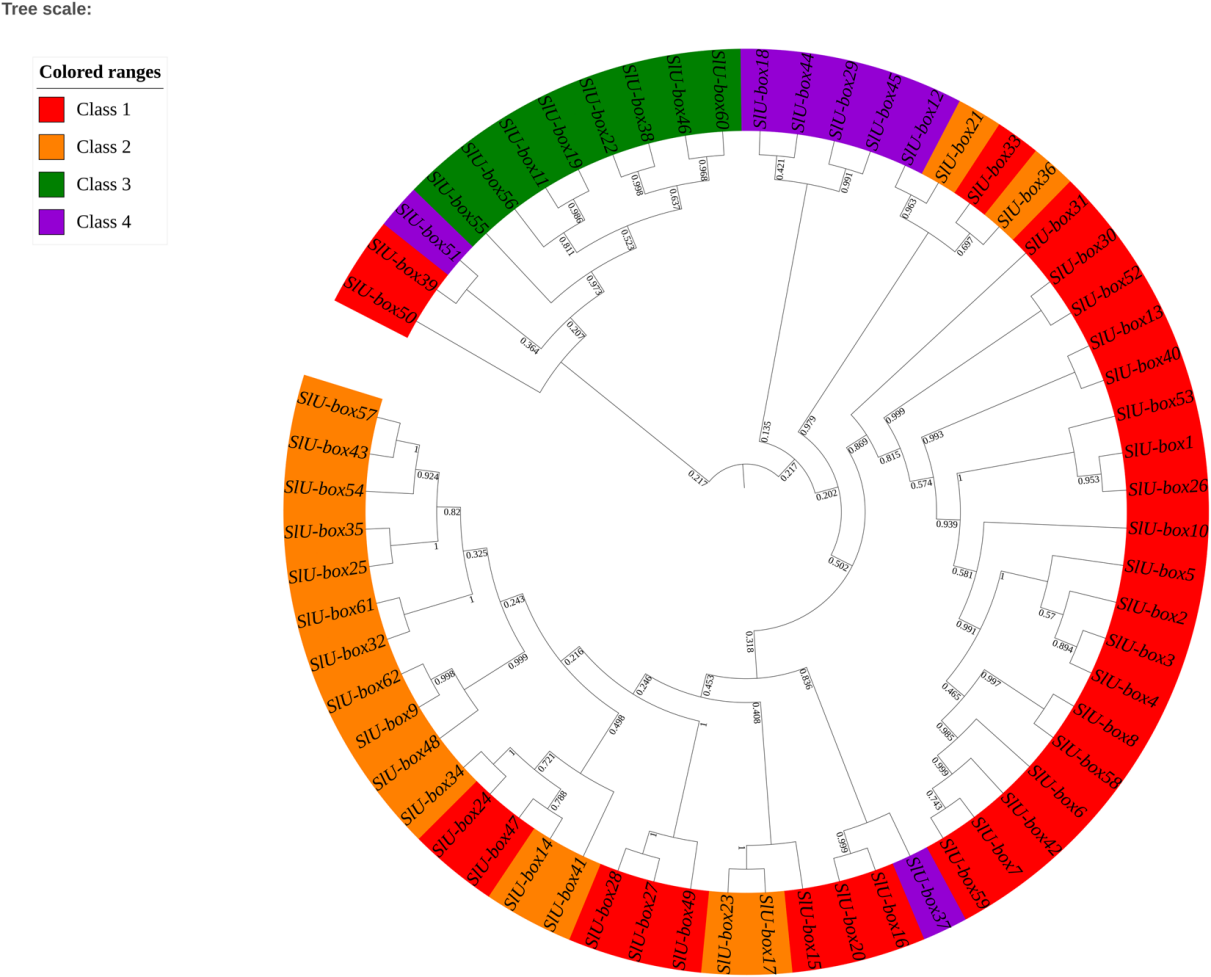
The phylogenetic tree of *Solanum lycopersicum* U-box E3 ubiquitin ligase gene family members constructed by the Neighbor-Joining method with 1000 bootstrap values. The tomato U-box gene family is divided into four different classes represented by different colors.

The identified U-box E3 gene family members were named SlU-box 1 to SlU-box 62 as per their chromosomal positions from chromosome 1 to 12 (Supplementary Table S1). We have distributed the U-box E3 family into four groups (Supplementary Figure S3), according to the presence of the U-box domain (Class 1), U-box domain with armadillo repeats (Class 2), U-box domain with protein kinase domain (Class 3) and U-box domain with other domains such as WD40, KAP, Ufd2P, TPR, and RPW8 (Class 4). To inquire about the relationship among tomato U-box E3 family members, a phylogenetic tree was constructed using the maximum likelihood method with 1000 bootstrap replicates (Fig. 3). The largest group was Class 1 with 30 genes, then Class 2 with 17 genes followed by Class 3 and Class 4 with 8 and 7 genes, respectively. Class 3 U-box genes with armadillo repeats were found to be very similar to each other than any other class, irrespective of the presence of their numbers of domains in the structure. Except for SlU-box 55, all the genes in Class 3 were sharing significant similarity suggesting minimum evolutionary divergence among Class 3 members. Around 70% of the Class 1 genes were highly conserved and shown a great similarity with each other. The rest of the 30% members were distributed along with other classes and found somewhat similar to Class 2 and 4. SlU-box 15, 24, 27, 28, 47, and 49 genes of Class 1 were closely related to Class 2 members while SlU-box 39 and 50 were close with Class 4 members. The classes are based on the presence of the particular functional domain in addition to core the U-box domain. It is interesting to observe the evolutionary organization of few Class 1 members with all other classes because Class 1 possesses only the U-box domain yet adjoining other class members. Furthermore, SlU-box 37 of Class 4, unlike other Class 4 genes, was showing less similarity with its group but was clustered with Class 1 members. Surprisingly, the SlU-box 51 of Class 4 and SlU-box 39 and 50 of Class 1 were clustered with Class 3 members indicating conservation among the diverse domain-containing SlU-box E3 members. These events particularly suggest that few members of the gene family expanded with fragmentary functional domains therefore grouped based on the core domain. The chromosomal organization of the U-box E3 genes also suggests the evolutionary interconnections between all the identified genes (Supplementary Figure S4). It was observed that U-box E3 genes were highly clustered on chromosomes 1, 4, and 5 while no gene was present on chromosomes 8 and 10. Most of the genes were located on distal regions of the chromosomes. It also indicates the gene evolution plausibility of the U-box E3 gene family due to its distal region location. Chromosomal recombination is the fundamental event for the expansion and divergence of gene families and non-coding regions. The end regions of the chromosomes, due to high rates of recombination, are preferable sites for gene evolution and are associated with duplication and divergence^43^. Therefore, we hypothesize that the gene family may have acquired new domains in addition to the core U-box domain to participate in a specific cellular mechanism through ubiquitination. The expansion of the gene family could be a result of the gain of new function in tomato.

### Gene duplication and synteny analysis

We have observed that around 30.64% of the identified U-box E3 ubiquitin ligase genes participated in gene duplication events in the *Solanum lycopersicum* genome (Supplementary Table S5). A total of sixteen events of duplication were recorded among the U-box E3 gene family. The gene duplication was found on one or two loci. The synteny analysis revealed that SlU-box 42, 43, 46, 57, and 61 were duplicated on two loci while remaining candidates were found on one locus. The Ka/Ks (non-synonymous/synonymous substitution ratio) is vigorously used for the identification of the nature of duplication and evolutionary pattern in the genome^44,45^. The duplicated U-box E3 gene family members in tomato have shown the Ka/Ks ratio, less than 1 which indicates the dominancy of purifying selection. Except for SlU-box 54, which was observed with intra-chromosomal segmental duplication, all other members showed inter-chromosomal duplication^46^. The gene family members were scattered throughout tomato chromosomes that could be under the influence of segmental duplications. Most of the Ka/Ks ratio values were laid from 0.1 to 0.31 that suggests the removal of deleterious mutations/alleles throughout the U-box E3 gene family as a result of negative/purifying selection^47^. The maximum value was 0.3176 for SlU-box 11 and the minimum was 0.1166 for SlU-box 54. We also found four loci with null Ka/Ks value. The syntenic regions were identified when the distance between two adjacent matches was 100 kb. This method captured high-resolution small-scale gene rearrangement events. We found a minimum of six anchors and a maximum of thirty-three anchors in the syntenic regions which defines the intensity of the duplication event (Supplementary Figure S6). The syntenic analysis revealed ten homologous gene pairs in the U-box E3 gene family. Our results show conserved syntenic regions distributed in the inter-chromosomal regions. The expansion of gene family could be a result of linkage specific genome duplication. The gene duplication findings imply that U-box E3 ubiquitin ligase gene family has undergone purifying selection, a process of eradicating deleterious mutations that can resist extreme distinction among the gene family members while conserving the core functional domains^48^. The synonymous substitutions per site also propose the evolutionary timescale for the whole genome duplication events. Our data revealed the range of synonymous substitutions per site between 0.16 and 0.51. The duplications of the U-box E3 ubiquitin ligase gene family in tomato apparently derived from recent whole-genome duplication events estimated 30–45 million years ago^49^. Taken all together, the results predict the segmental duplication as a leading factor for the U-box E3 gene family extension that could effectively contribute to the preservation of the structures of the genes as well as functions. It can also be a factor behind the acquisition of new functional domains on the genes.

### Promoters and gene ontology analysis

The promoter region of the identified 62 tomato U-box E3 genes was analyzed using PlantCARE database^50^ for the presence of the cis-regulatory elements (Supplementary Table S7). The cis-regulatory elements, the binding sites for transcription factors, carry information to regulate the gene expression in certain environmental responses or biological pathways. The occurrence frequency of the elements was represented as a word cloud image (Supplementary Figure S8). We found jasmonic acid-responsive elements (TGACG, CGTCA), MYB binding site involved in drought induction (TAACTG), gibberellin responsive elements (CCTTTTG,AAACAGA), endosperm expression (TGTGTCA), abscisic acid-responsive elements (CACGTG), ethylene responsive elements (ATTTCAAA), heat-responsive elements (AAAAAATTTC), defense and stress-responsive elements (ATTTTCTTCA), circadian control elements (CAANNNNATC) abundantly in U-box E3 family. The presence of these elements predicts the notable participation of U-box E3 gene family members in hormonal pathways and stress responses. We have noticed jasmonic acid, drought inductive, endosperm expression, defense, stress-responsive and abscisic acid-responsive elements with the highest frequency compared to other elements in the U-box E3 gene family. It suggests the critical requirement of the U-box E3 ubiquitin ligase in various cellular pathways. However, we could not observe any definite pattern of cis-element appearance with the four classes of the U-box E3 gene family. A few genes were also noticed with the shoot (GATAatGATG) and root (TGACGTCA) specific elements, cell cycle regulation (CCCAACGGT), seed-specific element (CATGCATG) and alpha-amylase conserved elements (TATCCATCCATCC). The presence of a significant number of the regulatory elements suggests strong participation of the U-box E3 gene family in plant development and responses under abiotic and biotic stress conditions. The data also supports the active involvement of the U-box E3 gene family in hormonal regulatory pathways.

Gene ontology analysis (Supplementary Figure S9) of the identified U-box E3 gene family revealed that most of the genes were involved in the metabolic (GO: 0008152) and cellular (GO: 0009987) processes. The U-box E3 gene family cellular component was predicted to be present in the cytoplasm (GO: 0005737) and nucleus (GO: 0005634). The proteins could be classified based on the gene ontology analysis into cell adhesion molecules (PC00069), cytoskeleton protein (PC00085), enzyme modulators (PC00095), signaling molecules (PC000207) and storage proteins (PC00210). It predicts the participation of the U-box E3 gene family in various biological processes. The gene ontology and promoter analysis together put up strong evidence for the association of tomato U-box E3 gene family in various growth and development operations.

### Gene expression analysis in vegetative and reproductive tissues

**Figure 4:**
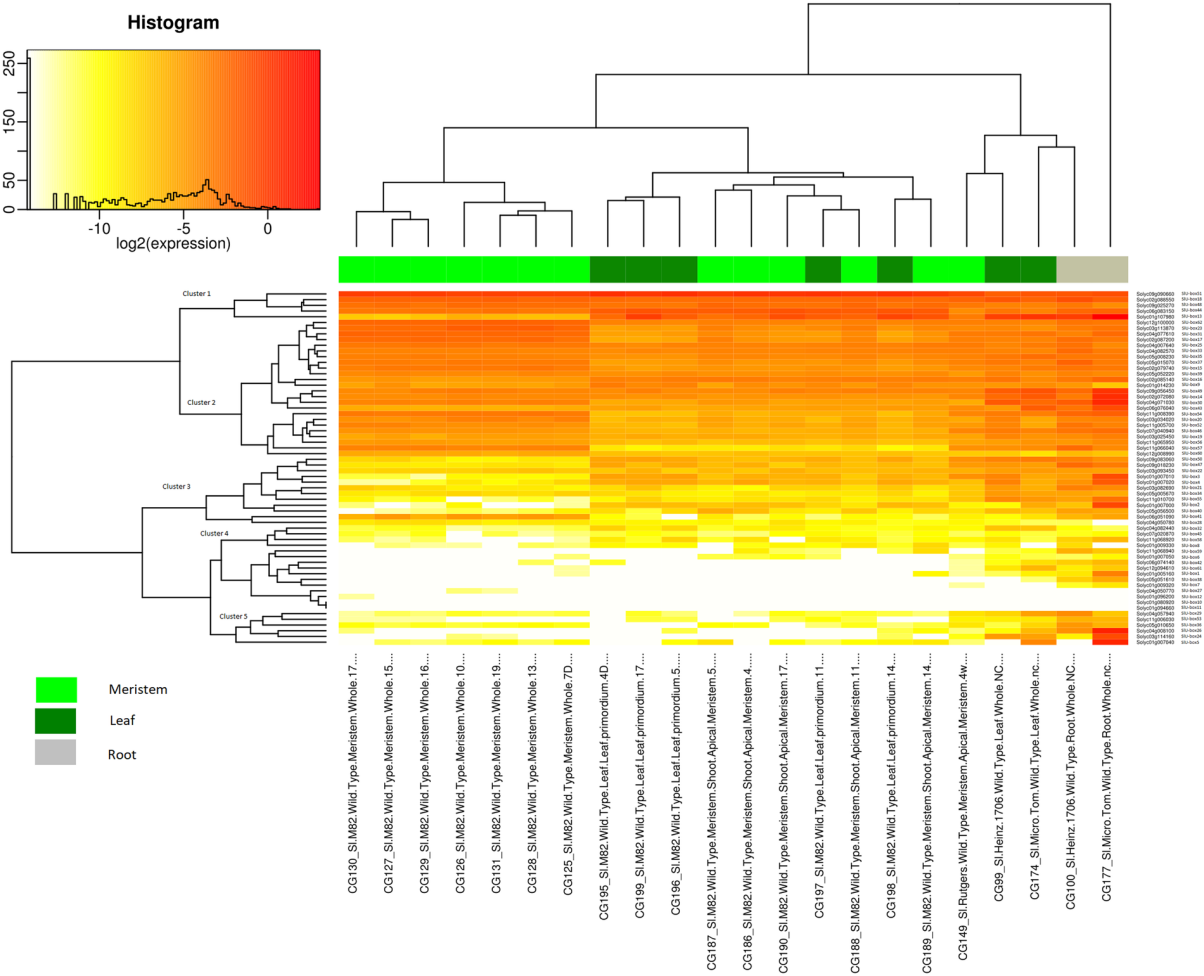
The gene expression level of the 62 U-box gene family members in vegetative tissues (leaf, meristem, root, and apical meristem) was represented by a heat map. (For detailed conditions refer Supplementary Table S10).

**Figure 5:**
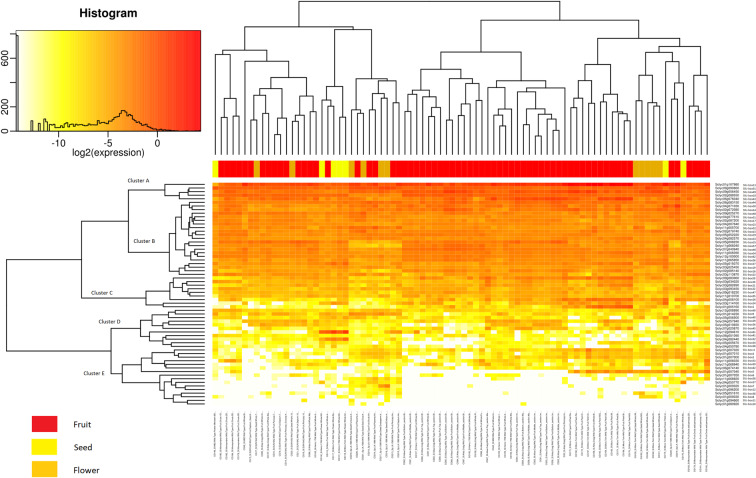
The gene expression level of the 62 U-box gene family members in reproductive tissues (flower, fruit, and seed) was represented by a heat map. (For detailed conditions refer Supplementary Table S11).

The Tomexpress database was used for the study of the gene expression of the U-box E3 gene family in tomato^51^. The gene expression analysis was analyzed in both vegetative (Fig. 4) and reproductive tissues (Fig. 5). The vegetative tissues (leaf, meristem, root and, apical meristem) were observed for the expression level of the U-box E3 gene family (Supplementary Table S10). It was observed that Cluster 1 genes were uniformly expressed in all the stages of meristem tissues (EVM, MVM, LVM, LM, TM and, SIM) except SlU-box 13 which had comparatively lower expression in whole tissue. Similarly, Cluster 2 genes were also adequately expressed in meristem tissues and the intensity was comparable with Cluster 1 genes. This suggests the active role of U-box E3 genes in the regulation of asymmetric cell division and cell proliferation in tomato meristem tissues^52^. The Cluster 3 genes mostly exhibited lower expression except for SlU-box 41 which moderately appeared in the meristem tissues. The Cluster 4 and 5 were found with low or no expression level. Cluster 1 and 2 genes emerged with an uplifted level of gene expression in leaf compared to Clusters 3, 4, and 5 genes. A contrasting expression of SlU-box 51 and 13 genes was noted in leaf tissues. In apical meristem tissues, the Cluster 1 genes were reported with the highest level of expression and Cluster 2 genes with a moderate level of expression. Interestingly, we could observe an exceptional expression of SlU-box 2, 3, 5, 13, 14, 24, 25, 30, and 49 in root tissues than other genes while Cluster 4 genes showed a weak expression profile. Similar gene expression profiles were observed in *Arabidopsis thaliana*, where AtPUB60 (At2g33340) and AtPUB49 (At5g67530) were highly expressed in root tips^20^. Moreover, U-box containing armadillo repeats were not found in root tissues of *Arabidopsis thaliana*, while our results exclusively show a strong pattern of SlU-box 14, 25, and 43 carrying armadillo repeats revealing the intervention of the U-box E3 ubiquitin ligase in tomato plant growth and development. Most of the strongly expressed genes in root tissues were belonging to Class 1 asserting the crucial association of the U-box domains in tomato vegetative tissue development.

The gene expression profiles of the reproductive tissues were studied to gain an understanding of the connection of the U-box E3 gene family in tomato plant growth and development (Supplementary Table S11). Cluster A genes were dominating the expression level in flower, fruit, and seed tissues followed by most of the Cluster B and few of Cluster C genes (Fig. 5). The flower tissues were found with moderate expression compared to fruit tissues. Cluster A and B along with SlU-box 61 and 32 were highly expressed in the flower buds suggesting the importance of U-box E3 ligase in flower bud development. In *Arabidopsis thaliana*, U-box gene56 (At1g01670), found to be highly expressed in flower buds which support our results^20^. Interestingly, the SlU-box 61 and 32 were featured with armadillo repeats indicating domain-specific participation in flower development. The U-box E3 genes containing armadillo repeats were previously observed to be associated with flower development in *Arabidopsis thaliana*^27^. Similarly, Cluster A and B genes were dominating the seed embryo, seed endosperm, and seed coat tissues while a majority of the U-box E3 genes were expressed in the immature green, mature green and breaker, orange, and red stage seed tissues. The gene expression of Cluster C, D, E showed remarkable variation in fruits and flower tissues of *Solanum pimpinellifolium*, that is closest to *Solanum lycopersicum* and frequently used for breeding. The U-box E3 gene family could be among the factors that leads to the fundamental difference in fruits and other reproductive organs of these species. The U-box E3 gene expression in reproductive tissues was independent of our domain-based classification. Therefore, we could observe the amalgam of all four classes expressed in various tissues. A variety of the conditions (ovule, pericarp, septum, flesh, peel, mature and immature fruits, breaker, orange, red and ripe) were selected to observe the relationship of the U-box E3 gene family in the physiological development of fruit tissues. It was observed that SlU-box 13, 43, 50, and 51 were highly expressed genes that are strongly predicted to have a role in fruit development and ripening in tomato. SlU-box 1 and 26 genes from Cluster 3 were found to be significantly expressed in fruit peel and flesh tissues. SlU-box 5 from Cluster 5 was observed exclusively in fruit flesh, peel, mature green fruit, immature green fruit, pericarp and seed tissues only. Overall, the Cluster 1, 2, and 3 genes were highly expressed in fruit tissues. Our results predict the strong association of the U-box E3 ubiquitin ligases in tomato plant growth and development evidenced by transcript expression profiles and promoter study.

### Gene expression analysis under abiotic, biotic stresses and hormone treatment

**Figure 6:**
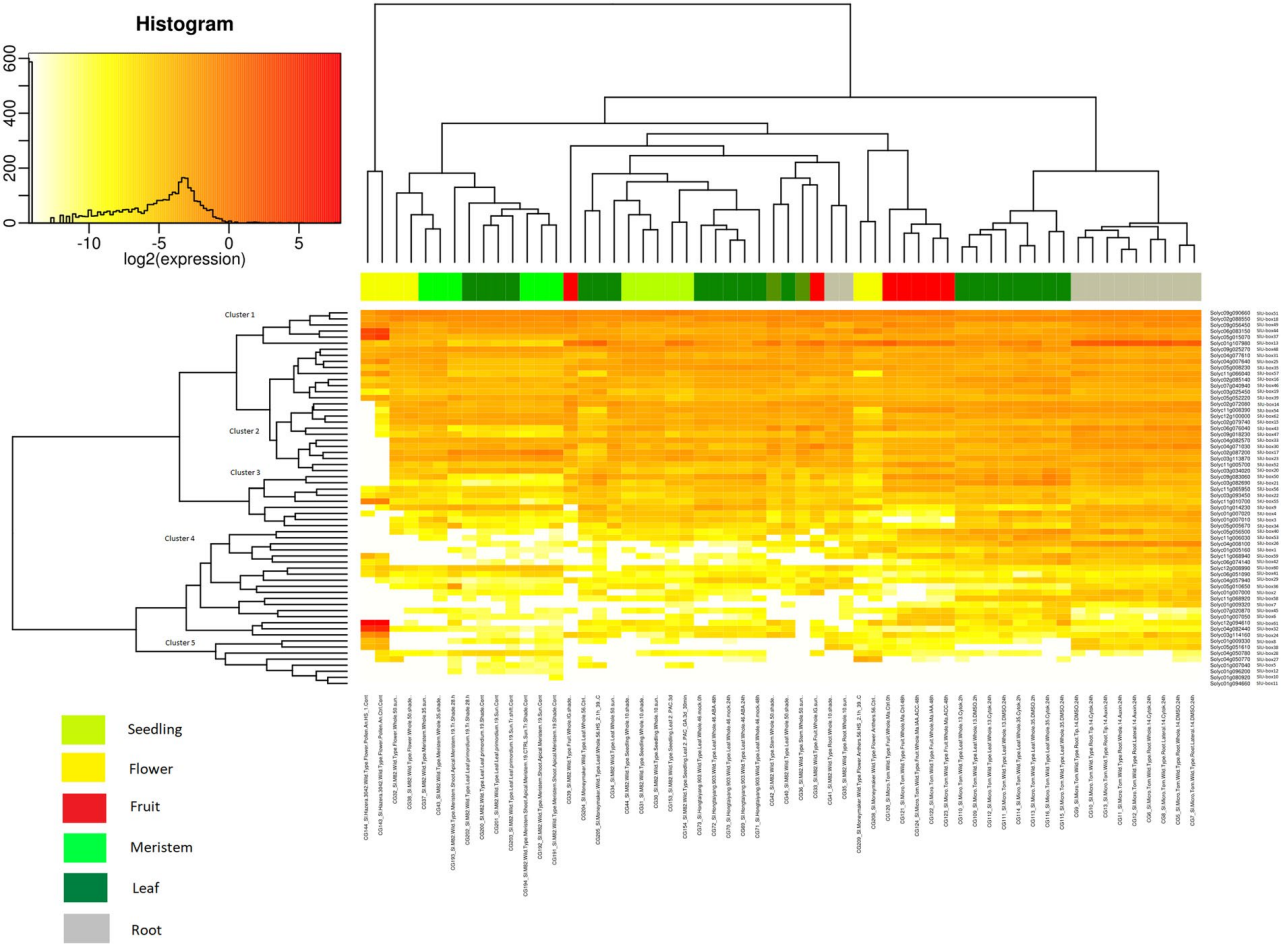
The gene expression level of the 62 U-box gene family members under abiotic stress and hormone-treated vegetative and reproductive tissues is represented by a heat map. (For detailed conditions refer Supplementary Table S12).

The gene expression was analyzed for abiotic stress conditions (sun, shade and, heat shock), hormone treatment (auxin, cytokinin, abscisic acid and, gibberellin) and biotic stress (Tomato Yellow Leaf Curl Virus, Virus-Induced Gene Silencing of Argonaute genes, *Funneliformis mosseae* and *Meloidogyne javanica*), as mentioned in Supplementary Table S12 and Supplementary Table S13, respectively. We could observe significant expression patterns among all the U-box E3 gene clusters in root, leaf, stem, and fruit tissues under hormonal treatment compared to untreated samples (Fig. 6). A little or no expression was found in most of the Clusters 4 and 5 genes. The SlU-box 13 and 26 were highly expressed in root tissues under auxin and cytokinin treatment. The expression of SlU-box 41 was increased in auxin treated root tissues when compared to untreated and cytokinin treated root tissues. The SlU-box 13 expression was also dominating in fruit tissues under sun and shade treatment as well as IAA and ACC hormone treatment. The gene expression variation was found in Clusters 4 and 5 genes under auxin and cytokinin treatment in root tissues. There was no major expression difference among the U-box E3 gene family under cytokinin and ABA treatment as well as the heat shock treatment in leaf tissues. The AtPUB19 was noticeably negatively regulating the ABA responses in Arabidopsis^30^. However, a moderate expression was observed in Clusters 1, 2, and 3 on short and prolonged exposure of ABA hormone. A little difference in expression profiling under Gibberellins and Paclobutrazol treatment was observed mostly in Clusters 4 and 5 genes. In meristem tissues, the expression was increased in 35 DPG sun and shade treated tissues compared to sun and shade control tissues (Fig. 6). SlU-box 6, 37, 44, and 61 were highly expressed in flower pollen tissues but significant differences in expression could not be noticed. SlU-box 9, 14, 15, 27, 28, 29, 43, 54, and 62 genes were not expressed under heat shock in flower pollen tissues when compared to control pollen tissues where the expression was observed. SlU-box 4 was expressed under heat shock conditions in the flower pollen tissue. It was also observed earlier where *Arabidopsis thaliana* U-box containing E3 ubiquitin ligase AtCHIP, was identified with a role in stress responses. However, the transcript expression did not increase with increased stress. The prolonged heat shock could not induce any significant expression difference in flower anther tissues. The expression profiles manifest a vital picture of U-box E3 ubiquitin ligase engagements in abiotic stress conditions.

**Figure 7:**
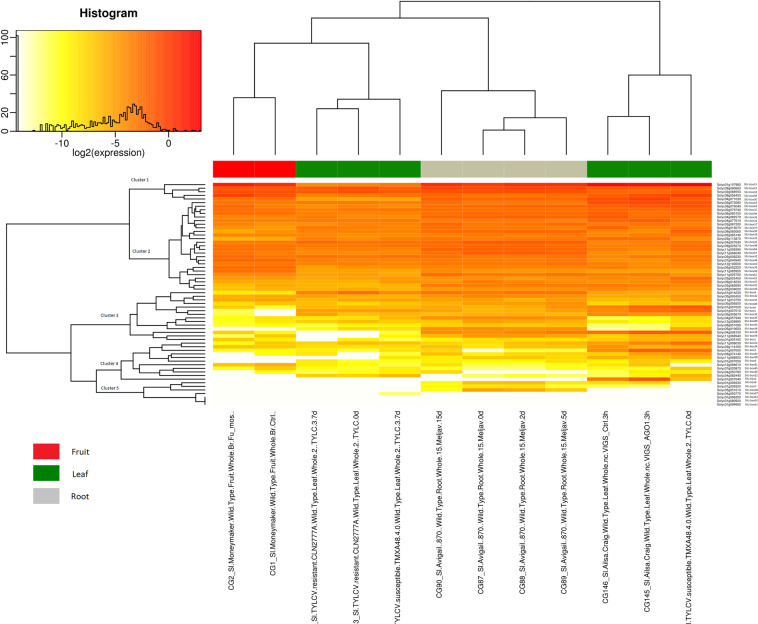
The gene expression level of the 62 U-box gene family members under biotic stress treated tissues is represented by a heat map. (For detailed conditions refer Supplementary Table S13).

The fruit, leaf, and root tissues treated with pathogens were analyzed for the U-box E3 ubiquitin ligase gene expression (Fig. 7). The root tissues treated with *Meloidogyne javanica* have observed with significant expression level U-box E3 genes. SlU-box 2 and 32 were expressed profoundly in 2- and 5-days infected root tissues that further increased in the 15 days infected tissues compared to null expression in the control sample. SlU-box 28 did not express in the control and short incubation but only expressed in 15 days infected tomato plant root tissues. It suggests that SlU-box 28 could be activated upon longer exposure to infection and participating in later stages of pathogen response. SlU-box 8, 22, 25, 38, 40, 47, 57, 58, 59, 60 were downregulated while SlU-box 3, 4, 14, 23, 28, 42, 45, 49, 56, 61 were upregulated in 15 days incubated tomato root tissues treated with *Meloidogyne javanica*. Fruit tissues infected with *Funneliformis mosseae* for the experiment and it was found that SlU-box 1, 3, 26, 34, 43, 49 were upregulated and SlU-box 28, 40, 42, 53, 59 were down-regulated in fruit tissues. Leaf tissues infected with TYLCV resistance observed with SlU-box 1, 13, 34, and 40 were up-regulated while most of the genes were not influenced. The TYLC susceptible leaf tissues were noticed either with little or no difference or low expression compared to control in most of the U-box E3 gene family. It was comparable to rice U-box E3 genes where OsPUB4, 12, and 23 were expressed in *Magnoporthe oryzae* resistance and susceptible plant and OsPUB51, 64, and 73 were found with stronger expression in susceptible plants^24^. SlU-box 2, 3, 4, 5, and 30 were observed with high expression in VIGS of AGO1 (Argonaute1) ortholog in leaf tissues. SlU-box 6, 21, 33, 37, 39, 42, 44, 60 and 62 were down-regulated to VIGS treated leaf tissues (Fig. 7). We can observe the activation of U-box E3 ubiquitin ligase gene expression in most of the treatments. Upon pathogen exposure, the U-box E3 ligases were observed to disrupt the proteins related to translocation, internalization, and, perception^53,54^. The active expression profile under biotic stress suggests the deeper involvement of the U-box E3 ubiquitin ligases in the tomato plants.

### Gene expression patterns in duplicated genes

**Figure 8:**
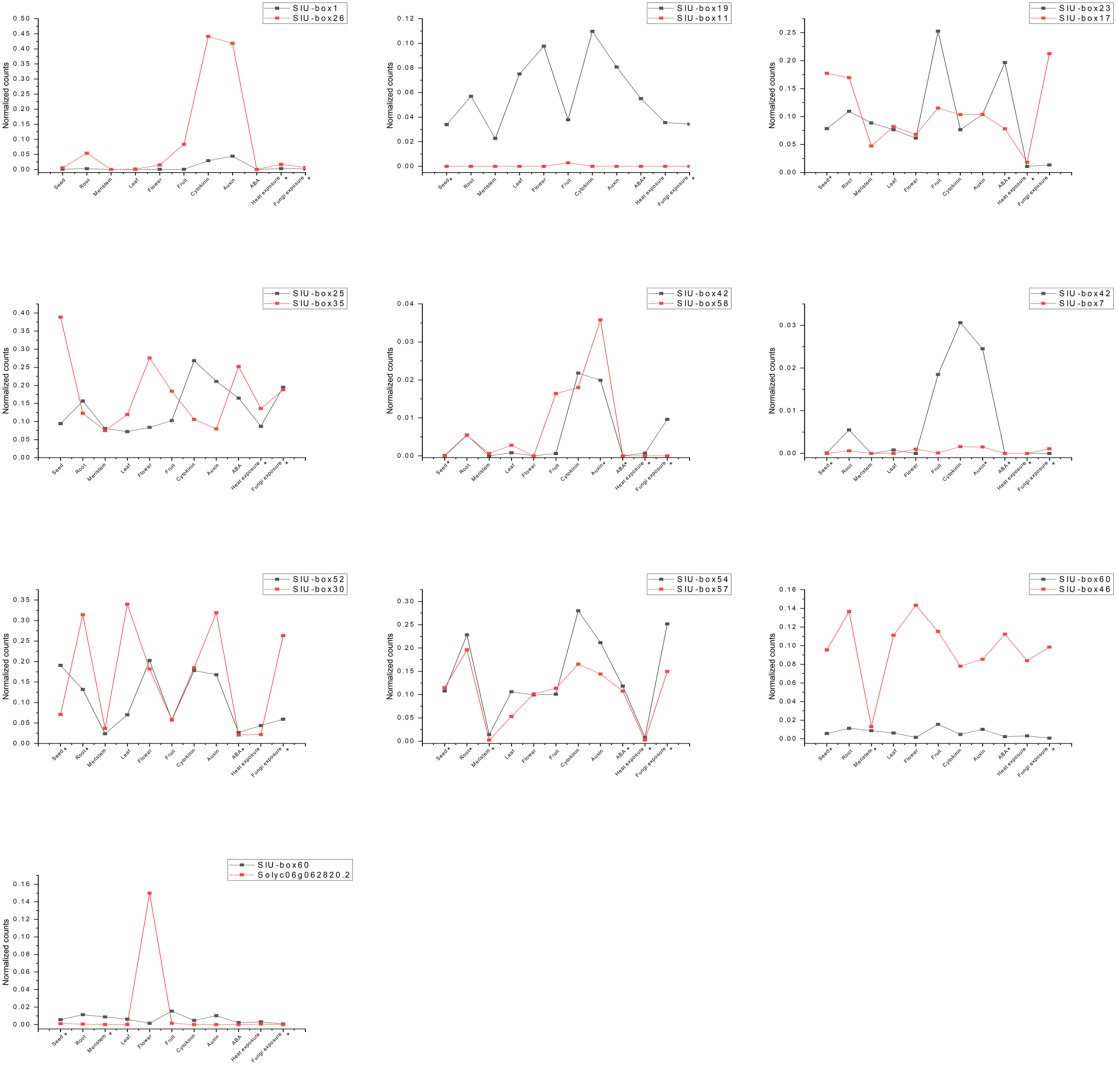
A comparative study of gene expression pattern between duplicated genes (total ten pairs) in seed, root, meristem, leaf, flower, fruit, cytokinin, auxin, abscisic acid (ABA), heat exposure (abiotic stress), and fungal exposure (biotic stress) treatments. The identified cis-elements conditions for the particular gene pairs are marked as (*).

The gene expression profiles of duplicated gene pairs, identified from syntenic analysis, were scrutinized to gain insight into functional stabilities under various growth stages and stress conditions (Fig. 8). Moreover, few representative conditions from RNA sequencing data with the predicted cis-regulatory elements were marked to inspect the functional conservation efficacy of the duplicated genes. The results reveal huge differences in the gene expression pattern among the duplicated genes. The SlU-box 11 and 7 duplicated from SlU-box 19 and 42, respectively did not express substantially in all the treatments suggesting pseudogenization after duplication. The pseudogenes undergo disruptive mutation leading to either distortion of regulatory sequences required for transcription events or defective splice junction while showing homology to functional genes^55^. The SlU-box 7 and 42, despite their predictive role in the heat and fungal stress, did not show significant expression. Interestingly, SlU-box 26 and 58 showed elevated transcript levels under cytokinin and auxin treatment conditions compared with their duplicated partners indicating subfunctionalization where the functionality of the original gene is distributed into two copies. Half of the gene duplications were estimated to be leading to functional divergence^56^. The specific upregulation during hormonal treatment could be a result of the complementation of partially divergent gene family members. The gene family duplication tends to accommodate the mutations and give rise to new functional features^57^. Similarly, SlU-box 30 and 46 transcript levels upraised in most of the treatments compared to their source genes. SlU-box 35 and 58 were selectively expressed in flower, ABA, and auxin treatments. Whereas, SlU-box 17 and 57 were showing transcript suppression under meristem, fruit, leaf, auxin, and ABA treatments. The disruption mutations are frequent under the influence of chromosomal rearrangements. These mutations give rise to pseudogenization that generates disabled copies of duplicated parental genes^58^. The treatments featured with cis-elements conditions manifested a large disparity not only validate the results but also propound the retention of the gene regulatory elements over the course of evolution. It can contribute to the divergence of gene families and the gain of new functions. Overall, our data represent striking evidence of functional divergence among duplicated U-box E3 gene family members in tomato.

## Discussion

E3 ubiquitin ligase is the largest family of the ubiquitin proteasomal degradation mechanism that regulates the ubiquitination of the substrates^5,59^. U-box E3 ubiquitin ligases are widely distributed in the plants and reported to participate in many cellular functions^38,59–63^. We have identified 62 tomato U-box E3 ubiquitin ligase genes with U-box domains utilizing *in-silico* analysis. The tomato U-box E3 ubiquitin ligases are largely hydrophilic that provides continuous hydrogen bond bridge for recurrent substrate interaction. The phylogenetic analysis of the tomato U-box E3 family showed a great similarity among all the four classes due to the presence of the core U-box domain in all the members. The expansion of the gene family was observed as a course of evolution where gene duplication and subfunctionalization of the native U-box domain in higher eukaryotes played a major role. Subfunctionalization is an alternative mechanism that leads to the retention of duplicated genes while partitioning the ancestral function. The augmentation of the gene family members could be a consequence of a neutral process of subfunctionalization^64^.

Gene duplication is a major factor for the expansion of the gene family over a period of time that is regulated by the environmental and biological factors in host organisms^45,65–68^. Gene duplication and syntenic analysis suggest the segmental duplication as a major force for the diversity in the tomato U-box E3 gene family. The syntenic analysis indicates the structural and functional conservation of the genes. The syntenic regions were found with numerous anchor genes associations. It allows the preservation of core functional groups and divergence as an extension to the duplicated gene. A significant level of conservation was found throughout the U-box E3 gene family. The gene duplication identified as the basis for the U-box E3 gene family divergence in tomato. The different domains added to the conserved region could have diversified the function of the U-box E3 ubiquitin ligase in the specific signaling. Both the inter-chromosomal and intra-chromosomal segmental duplication could be observed which led to 10 duplication events. It demonstrates that the SlU-box E3 gene family under the influence of subfunctionalization and neofunctionalization of duplicated genes resulted in the expansion of the gene family with diversity in structural organization and gene expression. The preserved sequences may undergo neofunctionalization over the time to devise divergence. The U-box E3 gene family in tomato was estimated to undergo two whole-genome duplication events around 36–82 million years ago and 148–205 million years ago^46,49^. The gene duplication is predicted to be responsible for the gaining of the novel functions in the tomato U-box E3 gene family. The structure and organization of the genes also indicate the diversity in a gene family among species^68^. The structural organization is related to the gene evolution and functional aspects of the gene family^68^. Many U-box E3 genes were either intronless or studded with three introns. A similar pattern of intronless genes of the U-box E3 gene family was also reported in Grapewine^69^. The U-box E3 genes bearing numerous introns could act as a mutational buffer that protects coding sequences from randomly occurring deleterious mutations^70^. The presence of the intron-less genes indicates the structural integrity among the U-box E3 family members. The analysis of the novel motifs provides a pattern of the nucleotide or amino acid sequences in a group of related sequences based on the position-dependent letter probability matrices^42,71^. The distribution of the identified 10 motifs among the tomato U-box E3 gene family suggests the structural and functional similarity among tomato U-box E3 genes. Motif 1 and 3 were found to be conserved and shown homology with the U-box domain. It also shows the presence of additional domains that may contribute to the complex structural formation in the U-box E3 gene family. Motif 2 was the exclusive feature of the Class 2 genes, which resembles the armadillo-like fold structure. The analysis aids in the evolutionary, structural, and functional prediction generated by the other experiments. The promoter analysis strongly predicts the association of the U-box E3 gene family in stress-related mechanisms, hormonal regulation, and development. The substantial difference in gene expression profiles of duplicated genes bearing common cis-regulatory elements could be a result of pseudogenization (Fig. 8). The presence of the various regulatory elements in the promoter region of the genes is vital evidence to support the participation of the U-box E3 gene family in the various tomato signaling mechanisms. The previous reports of the promoter analysis in the *Medicago truncatula* provide similar patterns of the elements that suggest a common association of the U-box E3 gene family in stress and hormonal regulatory pathways of plants^72^.

We have utilized the latest Tomexpress RNA sequencing database^51^ to generate a comprehensive understanding of the gene expression pattern of the U-box E3 gene family in different parts under various stress and hormone treatment conditions in tomato. The expression level in the meristem and leaf tissues of at least half of the genes were noted that strongly suggests the involvement in tomato vegetative tissue development. We could find exceptional gene expression of few U-box E3 genes in root tissues which indicate the participation of these genes in the root-specific mechanisms. Similarly, high-rise in U-box E3 gene expression was spotted in reproductive tissues such as fruit and flower suggesting the engagements of U-box E3 ligases in the crucial plant growth and development. The gene expression profiles, and cis-element prediction suggests the possible involvement of the U-box E3 gene family members in the flowering time and endosperm formation. The gene expression profile under abiotic stress and hormone treatment conditions show a variety of the expression of the U-box E3 genes in tomato. The U-box E3 ubiquitin ligases were reported to be involved in plant abiotic stress and hormone signaling^29,30,72^. The Cluster 4 and 5 genes expression were significantly altered under hormone and abiotic conditions in tomato. The data suggests both the positive and negative impact of U-box E3 genes in tomato. The findings support the previous studies where the U-box E3 gene was found to be negatively regulating the drought stress^23^ and ABA response regulation^29^. Further, the gene expression analysis also supports the promoter analysis of the U-box E3 gene family where we found significant elements involved in hormone regulation. The genes expression profiles validate the cis-regulatory elements prediction that makes it even more lucid to culminate the association of the U-box E3 gene family in the tomato development. The U-box E3 gene family members were reported to be expressing under biotic stress in grapevine^69^ as well as in tobacco and tomato^73^. The gene expression analysis under biotic stress in tomato suggests vital regulatory interventions of the U-box E3 gene family which is also supported by promoter study. The roots, leaf, and fruit tissues were noticed with altered U-box E3 gene expression under biotic stress. We predict a significant influence of the U-box E3 gene family on the biotic stress response in tomato. On the other hand, the duplicated genes detected with the altered gene expression add up to our findings and anticipate the preserved core function with substantial divergence leading to the expansion of the gene family. Our results imply that the U-box E3 gene family is an important clan of E3 ubiquitin ligase which is predicted to influence the development of tomato plant tissues as well as participate in the stress and hormonal pathways.

The requirement of such a large gene family for the plant system has not been understood so far. We can speculate that the variable expression of U-box E3 proteins in different tissues and the presence of novel protein interacting domains may qualify them to regulate many plant molecular mechanisms. We infer that U-box E3 proteins can not only respond to environmental stresses but also influence plant development. The E3 ubiquitin ligases are found to be regulating many cellular events such as stress responses, hormone signaling, cell division, senescence, and embryogenesis^26,38,60^. The characterization of U-box E3 ubiquitin ligases will provide a better insight into their roles in various cellular pathways. Our study predicts the significant participation of the U-box E3 gene family in the tomato plant development. U-box E3 ligases can be targeted for the development of an improved variety of crops with better immunity, yield, and stress tolerance. U-box E3 gene family is rarely studied in the tomato plant system and our analysis provides an overall picture of the U-box E3 gene family in tomato. It will serve as preliminary evidence for the study of the U-box E3 ubiquitin ligase enzymes in the tomato and other plant systems.

## Material and Methods

### Sequence retrieval and characterization

We have utilized the HMMER program for the identification of the potential U-box E3 ubiquitin ligase members in tomato. The profile HMM for U-box domains (PF04564) were retrieved from Pfam database^74^. HMM search was performed against the *Solanum lycopersicum* reference database (ITAG2.4) with default parameters and a significant *e*-value of 0.01^41^. The molecular weight, number of amino acids, aliphatic index, pI (iso-electric point), GRAVY (Grand average of hydropathicity), number of nucleotides were analyzed using ProtParam and ExPasy-Compute pI/Mw^75^. The subcellular localization was predicted using the TargetP1.1 server (http://www.cbs.dtu.dk/services/TargetP/)^76^. The chromosomal location, intron count, and sequence information were retrieved from Sol Genomic Network^77^, PANTHER^78^, and Phytozome database^79^.

### Gene structure and motif analysis

To identify and visualize the structural organization (introns, exons, and untranslated regions) of the tomato U-box E3 gene family, the GSDS (Gene Structure display System) tool was used (http://gsds.cbi.pku.edu.cn/)^80^. The novel conserved motifs in the tomato U-box E3 gene family were identified using a motif-based sequence analysis tool, MEME suite (http://meme-suite.org/)^71^. A total of 10 motifs and a width limit of 200 amino acids were used for the analysis with other default parameters.

### Phylogenetic analysis and chromosomal localization

The evolutionary characteristics of the U-box E3 ubiquitin ligase were analyzed by performing multiple sequence alignment in Clustal W with default parameters followed by the construction of the phylogenetic tree using full-length U-box E3 sequences. The Maximum Likelihood method based on the JTT matrix-based model with 1000 bootstrap replications and partial deletion was used for the generation of the tree in the MEGA 7.0 version (https://www.megasoftware.net/)^81^. The visualization of the tree was performed by the iTOL v3 tool (https://itol.embl.de/)^82^. The information related to all 62 U-box E3 ubiquitin ligase genes was obtained from the Sol genomics database. The identified genes were mapped on 12 tomato chromosomes using ArkMap version 2.0 software (http://bioinformatics.roslin.ed.ac.uk/arkmap/)^83^. The chromosomal coordinates the 12 tomato chromosomes were retrieved from Ensembl plant database^84^.

### Gene duplication and synteny analysis

The Plant Genomic Duplication Database (http://chibba.agtec.uga.edu/duplication/mcscan/) was used for the gene synteny analysis and non-synonymous and synonymous substitution calculation^85^. The duplication events were obtained from Plant Genomic Duplication Database. The gene synteny was identified using the MCScan algorithm which scans multiple genomes to identify putative homologous regions and align them using gene anchors^86^. The CLUSTALW alignments of protein sequences of the gene pairs were used to guide CDS alignments by PAL2NAL. The Ks (synonymous substitution) was calculated by implementing the Nei-Gojobori method in the PAML package version 0.8 (http://chibba.agtec.uga.edu/duplication/mcscan/).

### Promoter and gene ontology analysis

The 1000 bp promoter sequences excluding transcription start sites were retrieved from phytozome database^79^ and used for the prediction of the plant cis-acting regulatory elements. PlantCARE database was used for the identification of the elements in the promoters (http://bioinformatics.psb.ugent.be/webtools/plantcare/html/)^50^. The word cloud image was generated using WordArt tool (https://wordart.com)^87^. The ontology information was retrieved from the PANTHER database and visualized as pie chart^78^.

### Tissue-specific gene expression analysis

The expression profile of the tomato U-box E3 ubiquitin ligase was investigated using the latest RNA sequencing data pipeline of the TomExpress database (http://tomexpress.toulouse.inra.fr/)^51^. The expression was analyzed in vegetative (root, shoot, meristem, and leaf) tissues and reproductive (flower, seed, fruit) tissues. The abiotic stress (sun, shade, and heat shock) and hormone (cytokinin, auxin, abscisic acid, indole acetic acid, and 1-aminocyclopropane-1-carboxylic acid) treated seedling, root, leaf, meristem, stem, flower, fruit tissues were used for the study. The root, leaf, and fruit tissues were selected for the biotic stress, where TYLC (Tomato Yellow Leaf Curl Virus), VIGS (Virus-Induced Gene Silencing) of argonaute genes, *Funneliformis mosseae* and *Meloidogyne javanica* were used from TomExpress RNA sequencing data (http://tomexpress.toulouse.inra.fr/). The data was visualized using Heat maps generated by TomExpress database^51^.

## Supplementary information

Supplementary Information.

## Data Availability

All data generated or analyzed during this study are included in this published article (and its Supplementary Information files).
