# Supplementary material for: Genome-wide analysis of the U-box E3 ubiquitin ligase enzyme gene family in tomato
Source: Sci Rep. 2020 Jun 12;10:9581. doi: 10.1038/s41598-020-66553-1 (PMC7293263; doi:10.1038/s41598-020-66553-1)
Supplement: Supplementary file 1 — Supplementary Information. [file 41598_2020_66553_MOESM1_ESM.pdf]

Genome-wide analysis of the U-box E3 ubiquitin ligase enzyme gene family in tomato

Bhaskar Sharma[1*], Joemar Taganna[2]

[1]TERI School of Advanced Studies, 10 Institutional Area, Vasant Kunj, New Delhi-110070

[2]SciBiz Informatics, 2/F Unit 3 CFI Building, Maharlika Highway, Brgy. Guindapunan, Palo, Leyte 6501 Philippines

*Correspondence: bs211190@yahoo.com

Supplementary Information

Supplementary Table S1

Supplementary Table S2

Supplementary Figure S3

Supplementary Figure S4

Supplementary Table S5

Supplementary Figure S6

Supplementary Table S7

Supplementary Figure S8

Supplementary Figure S9

Supplementary Table S10

Supplementary Table S11

Supplementary Table S12

Supplementary Table S13

Sol genomic ID	Gene Name	Class	pI	Mol. Wt.	Cellular Localization	Intron	Instability Index	Aliphatic Index	Grand Average of Hydropathicity (GRAVY)	Amino Acid	Gene Size	Chromosomal Position
Solyc01g005160.2.1	SlU-box1	1	8.81	50151.45	Mitochondria	0	44.83	105.84	-0.086	450	1709	SL2.50ch01:137781-139489
Solyc01g007000.2.1	SlU-box2	1	9.04	45651.55		0	48.03	102.27	-0.022	409	1374	SL2.50ch01:1567768-1569141
Solyc01g007010.2.1	SlU-box3	1	9.12	45788.83		0	47.65	102.28	-0.046	408	1360	SL2.50ch01:1571358-1572717
Solyc01g007020.2.1	SlU-box4	1	9.1	45860.87		0	45.08	101.05	-0.06	408	1511	SL2.50ch01:1576697-1578207
Solyc01g007040.2.1	SlU-box5	1	8.31	20237.43		0	67.92	80.67	-0.357	179	1061	SL2.50ch01:1583708-1584768
Solyc01g007050.1.1	SlU-box6	1	8.15	47535.19		0	39.63	104.63	-0.076	421	1415	SL2.50ch01:1591040-3502283
Solyc01g009320.1.1	SlU-box7	1	8.35	47224.41		1	43.65	107.7	-0.017	418	1511	SL2.50ch01:3500773-3502283
Solyc01g009330.1.1	SlU-box8	1	8.49	48077.36		0	46.93	104.79	-0.009	426	1281	SL2.50ch01:3515178-3516458
Solyc01g014230.2.1	SlU-box9	2	5.59	88538.18		3	42.51	100.07	-0.138	811	3301	SL2.50ch01:11880408-11883708
Solyc01g080920.2.1	SlU-box10	1	7.84	46136.32		0	50.38	99.83	-0.05	409	1621	SL2.50ch01:80262171-80263791
Solyc01g094660.2.1	SlU-box11	3	5.7	100199.5	Mitochondria	8	50.36	81.52	-0.377	894	8822	SL2.50ch01:86104268-86113089
Solyc01g096200.2.1	SlU-box12	4	6.39	159879		6	43.75	100.53	-0.088	1428	9254	SL2.50ch01:87291000-87300253
Solyc01g107980.2.1	SlU-box13	1	6.47	45391.47		0	48.49	104.89	0.109	415	1561	SL2.50ch01:95361899-95363459
Solyc02g072080.1.1	SlU-box14	2	8.39	79255.5	Mitochondria	0	42.55	97.76	-0.001	724	2175	SL2.50ch02:41352040-41354214
Solyc02g079740.1.1	SlU-box15	1	8.61	40289.41		0	39.3	110.35	0.03	371	1116	SL2.50ch02:44186074-44187189
Solyc02g085140.2.1	SlU-box16	1	6.06	77802.94		3	42.88	94.89	-0.227	701	3953	SL2.50ch02:48115261-48119213
Solyc02g087200.1.1	SlU-box17	2	7.16	53067.88	Mitochondria	0	49.99	94.86	-0.153	481	1446	SL2.50ch02:49668259-49669704
Solyc02g088550.2.1	SlU-box18	4	5.24	59179.26		11	44.23	103.65	-0.234	512	9871	SL2.50ch02:50596079-50605949
Solyc03g025450.2.1	SlU-box19	3	5.82	88886.22		8	45.98	45.98	-0.404	787	8501	SL2.50ch03:2862272-2870772

Solyc03g034020.2.1	SlU-box20	1	6.88	78060.61		5	43.07	97.26	-0.138	704	4495	SL2.50ch03:5725105-5729599
Solyc03g082690.2.1	SlU-box21	2	5.38	111798.1		3	51.28	111.88	-0.031	1007	4805	SL2.50ch03:52612130-52616934
Solyc03g093450.2.1	SlU-box22	3	6	90586.2		8	40.18	90.16	-0.365	808	6103	SL2.50ch03:54738489-54744591
Solyc03g113870.1.1	SlU-box23	2	6.65	57811.67	Mitochondria	0	45.84	89.38	-0.27	528	1587	SL2.50ch03:63927887-63929473
Solyc03g114160.1.1	SlU-box24	1	6.56	76293.08	Mitochondria	0	39.07	103.53	-0.039	682	2049	SL2.50ch03:64176752-64178800
Solyc04g007640.2.1	SlU-box25	2	8.47	69929.36		3	46.68	104.82	-0.101	647	3382	SL2.50ch04:1318689-1322070
Solyc04g008100.1.1	SlU-box26	1	8.96	49962.8		0	45.37	105.59	-0.105	442	1329	SL2.50ch04:1757824-1759152
Solyc04g050770.1.1	SlU-box27	1	5.65	49800.87		1	40.75	107.47	-0.169	443	1748	SL2.50ch04:48255605-48257352
Solyc04g050780.1.1	SlU-box28	1	7.2	48368.35		1	39.45	111.81	-0.108	431	1357	SL2.50ch04:48259054-48260410
Solyc04g057940.2.1	SlU-box29	4	5.86	165856		14	51.5	90.29	-0.286	1482	6167	SL2.50ch04:55054298-55060464
Solyc04g071030.1.1	SlU-box30	1	8.49	47011.6		0	41.04	103.09	0.093	427	1284	SL2.50ch04:57882497-57883780
Solyc04g077610.2.1	SlU-box31	1	6.72	45774.67	Mitochondria	1	40.57	112.77	0.111	419	1844	SL2.50ch04:62559572-62561415
Solyc04g082440.2.1	SlU-box32	2	5.81	72960.28		3	39.17	101.16	-0.27	649	2800	SL2.50ch04:66090632-66093431
Solyc04g082570.2.1	SlU-box33	1	5.7	84751.79		2	35.54	106.19	-0.03	772	5036	SL2.50ch04:66213281-66218316
Solyc05g005670.1.1	SlU-box34	2	7.82	76074.46	Mitochondria	0	36.48	108	0.111	685	2058	SL2.50ch05:498016-500073
Solyc05g008230.2.1	SlU-box35	2	8.28	70932.26		3	43.78	101.18	-0.138	650	4379	SL2.50ch05:2622044-2626422
Solyc05g010650.2.1	SlU-box36	2	5.93	117283.9		4	44.59	103.74	-0.139	1046	4394	SL2.50ch05:4861481-4865874
Solyc05g015070.2.1	SlU-box37	4	5.31	84727.77		4	45.87	92.71	-0.251	767	5659	SL2.50ch05:9775132-9780790
Solyc05g051610.1.1	SlU-box38	3	6.51	93988.7		7	47.21	84.53	-0.425	839	3523	SL2.50ch05:61980713-61984235
Solyc05g052220.2.1	SlU-box39	1	4.95	45597.87		7	39.3	61.89	-0.76	417	8493	SL2.50ch05:62498939-62507431
Solyc05g056500.1.1	SlU-box40	1	5.45	45191.82		0	47.81	109.85	0.085	401	1206	SL2.50ch05:65769827-65771032

Solyc06g051090.1.1	SlU-box41	2	8.53	61397.11		0	42.94	110.28	-0.045	562	1689	SL2.50ch06:34208643-34210331
Solyc06g074140.1.1	SlU-box42	1	8.84	47715.13		0	43.44	109.07	0.003	420	1263	SL2.50ch06:45857406-45858668
Solyc06g076040.2.1	SlU-box43	2	5.47	72075.47		3	45.22	100.3	-0.196	661	5135	SL2.50ch06:47209976-47215110
Solyc06g083150.2.1	SlU-box44	4	7.62	31770.26		7	44.46	83.8	-0.528	276	4682	SL2.50ch06:48687538-48692219
Solyc07g020870.1.1	SlU-box45	4	5.68	124140.6		14	42.53	99.41	-0.155	1110	5539	SL2.50ch07:14344450-14349988
Solyc07g040940.2.1	SlU-box46	3	6.13	83589.8		8	49.81	95.92	-0.226	746	5657	SL2.50ch07:51194049-51199705
Solyc09g018230.1.1	SlU-box47	1	8.77	75766.09	Chloroplast	0	42.43	103.31	-0.027	688	2067	SL2.50ch09:13637609-13639675
Solyc09g025270.2.1	SlU-box48	2	5.93	82014.43		3	40.09	95.49	-0.258	749	5387	SL2.50ch09:72441024-72446410
Solyc09g056450.2.1	SlU-box49	1	8.49	49907.72		1	43.23	104.3	-0.227	449	2132	SL2.50ch09:49222360-49224491
Solyc09g083060.2.1	SlU-box50	1	5.35	64108.22	Secretory	3	48.35	99.27	-0.237	574	4424	SL2.50ch09:68734004-68738427
Solyc09g090660.2.1	SlU-box51	4	6.12	56331.6		17	28.95	83.7	-0.236	521	8098	SL2.50ch09:70113114-70121211
Solyc11g005700.1.1	SlU-box52	1	8.76	46790.19		0	39.68	99.98	-0.033	420	1263	SL2.50ch11:547491-548753
Solyc11g006030.1.1	SlU-box53	1	8.47	48921.53		0	37.82	99.44	-0.026	430	1293	SL2.50ch11:840424-841716
Solyc11g008390.1.1	SlU-box54	2	5.53	68021.08		3	42.31	106.15	-0.188	624	2677	SL2.50ch11:2592403-2595079
Solyc11g010700.1.1	SlU-box55	3	6.73	91628.37		7	48.01	88.6	-0.406	798	4649	-SL2.50ch11:3735958-3740606
Solyc11g065950.1.1	SlU-box56	3	7.2	95735.26	Chloroplast	6	52.24	90.34	-0.208	855	10293	SL2.50ch11:51562211-51572503
Solyc11g066040.1.1	SlU-box57	2	6.04	65367.6		3	42.64	96.15	-0.282	603	7487	SL2.50ch11:51713001-51720487
Solyc11g068920.1.1	SlU-box58	1	8.85	45612.36		0	48.61	108.56	-0.076	404	1215	SL2.50ch11:53482855-53484069
Solyc11g068940.1.1	SlU-box59	1	7.54	47815.91		0	42.53	110.17	-0.016	423	1272	SL2.50ch11:53492962-53494233
Solyc12g008990.1.1	SlU-box60	3	7.57	85567.21		8	46.89	95.43	-0.325	760	4946	SL2.50ch12:2291562-2296507
Solyc12g094610.1.1	SlU-box61	2	5.4	60419.1		3	46.95	99.68	-0.348	537	3036	SL2.50ch12:64780504-64783539

Solyc12g100000.1.1	SlU-box62	2	5.77	89290.22	Secretory	3	40.36	98.88	-0.098	819	2873	SL2.50ch12:66914193-66917065

Supplementary Table S1: The classes, iso-electric point (pI), molecular weight, subcellular localization, introns, instability index, aliphatic index, Grand Average of Hydropathicity (GRAVY), length of gene, numbers of amino acids and chromosomal localization is given in the table.

MOTIF	WIDTH	BEST POSSIBLE MATCH
1	41	PPYFRCPISLELMKDPVTVATGQTYDRESIEKWLDSGNNTC
2	41	AIARAGAIPPLVDLLRSGSPRGKEDAATALFNLSINDENKG
3	21	LTHLDLTPNHTLRRLIQEWCE
4	80	QQATKAALQVLVNACPWGRNRIKAIEAGAIFELIELLLDSSEKRVCELMLILLDQLCTLAEGRAELLNHPGGLAIVSKKI
5	96	FTFEEIETATSSFSESLKIGEGGYGTVYKGELHHTPVAIKVLHSEASQKPEEFQQEVEILSKLHHPHLVTLLGACPESGCLVYEYMENGS LEDRLL
6	41	VLQEMLRVGVVSKLCLVLQVDCSEKTKEKARELLKLHSKVW
7	100	TEYRDSDPAGTFAYMDPEYQRTGTLTPKSDVYAFGIILLQLLTARRANGLVHEVEEAIDKNNLVNVLDPSAGDWPLEETKELAKLALK CCELRSRDRPDL
8	100	HGIERFPTPKPPVSKPQIIKLLKEAKSPKLQMKSLKRLRSIASENDANKRCMESAGAMEFLASIINKNSNEVFEEEEGFMSIKDEALSILY QLKLSENGL
9	21	PLVELLQNGTSRAKRKAAKLL
10	29	RKLSSGSLEVQRKAAAELRLLAKRNMDNR

Supplementary Table S2: Ten motifs identified using MEME suit for U-box gene family

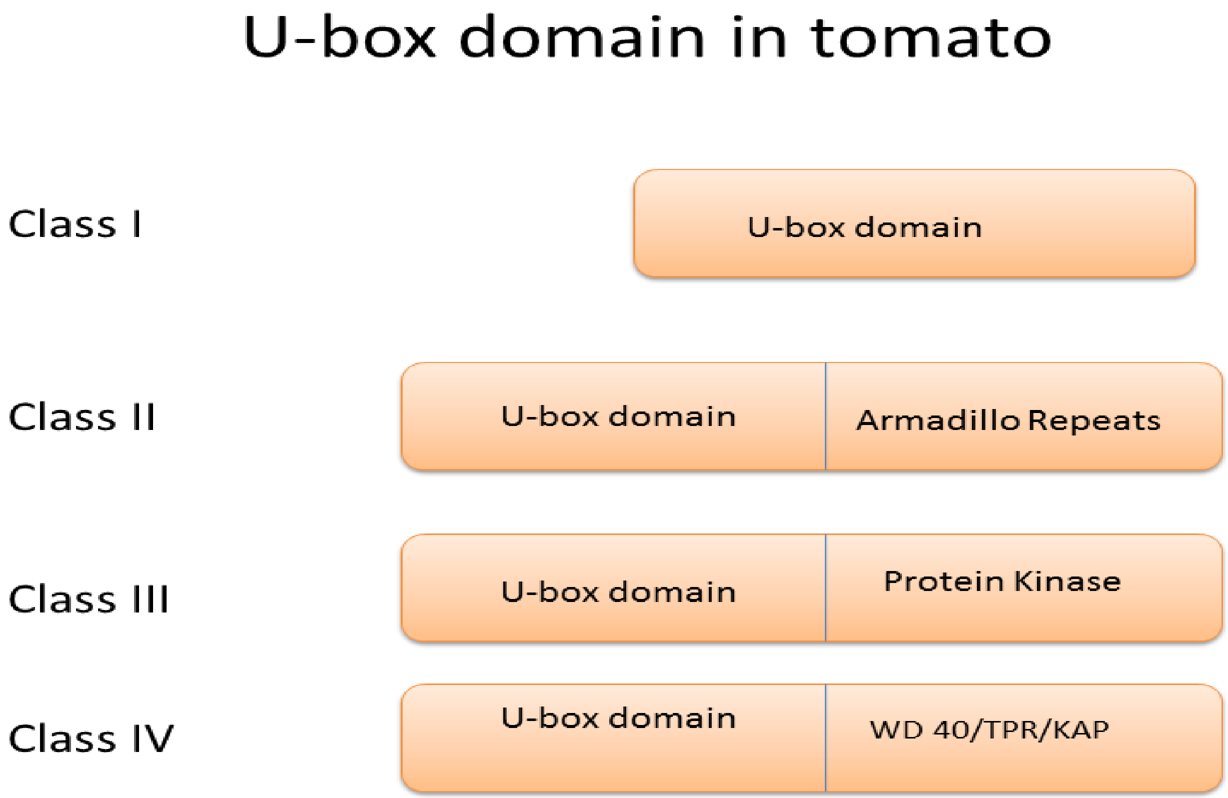

Supplementary Figure S3: A diagrammatic representation of the classes of tomato U-box E3 ubiquitin ligase enzymes

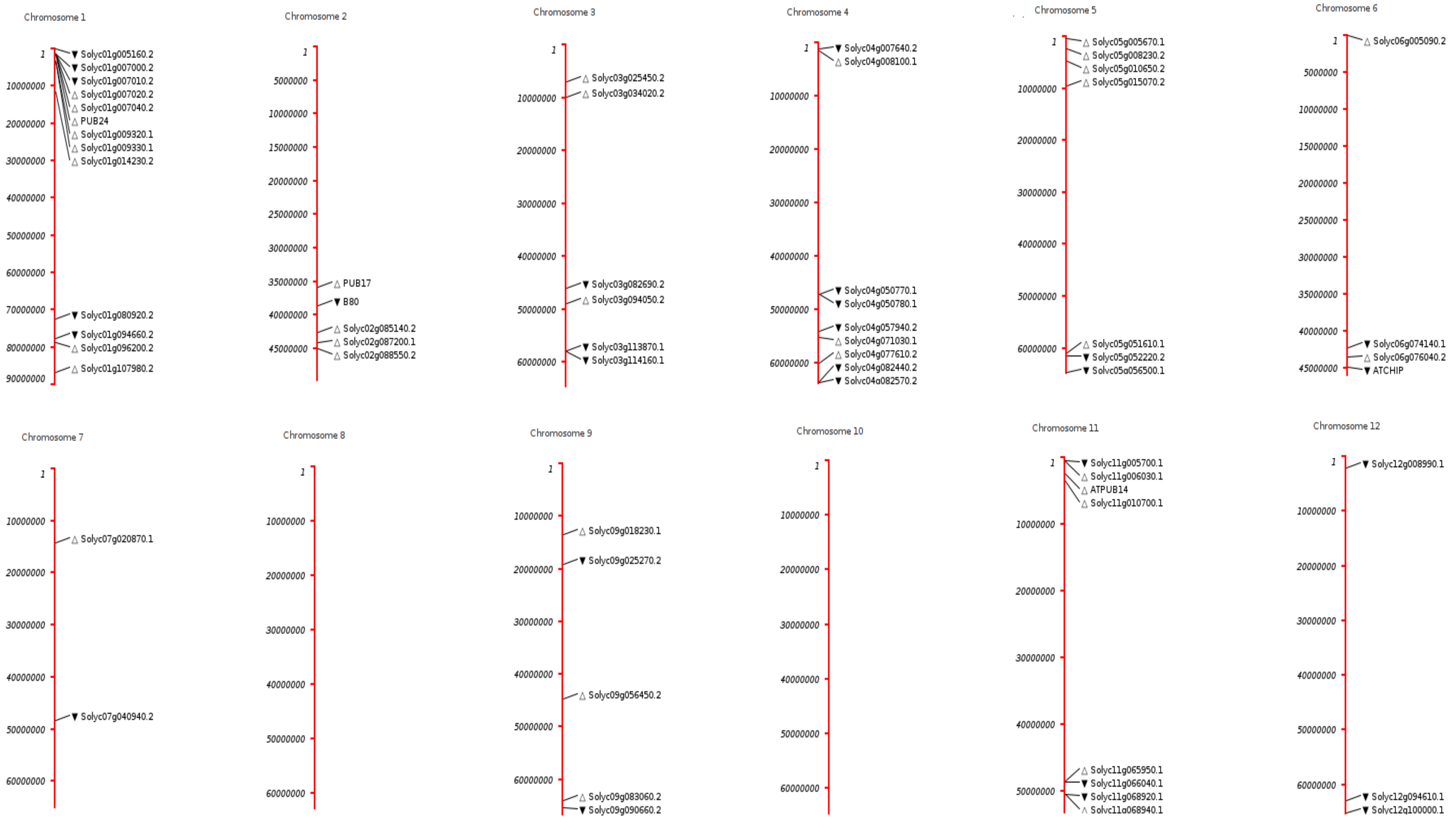

Supplementary Figure S4: The diagram represents chromosome map of the tomato genome constructed by Arkmap. The identified 62 U-box gene family is localized on 12 chromosomes.

Anchors	BLOCK_SCORE	E_VALUE	LOCUS_1	LOCUS_2	Ka	Ks	Ka/Ks
**8**	297	2E-142	Solyc01g005160.2	Solyc04g008100.1	0.27	0.85	0.31764706
**8**	290	3E-35	Solyc01g094660.2	Solyc03g025450.2	0.51	2.06	0.24757282
**16**	572	2E-31	Solyc02g087200.1	Solyc03g113870.1	0	0	0
**33**	1225	3E-61	Solyc04g007640.2	Solyc05g008230.2	0.16	0.58	0.27586207
**32**	1210	5E-140	Solyc06g074140.1	Solyc11g068920.1	0.5	1.65	0.3030303
**14**	511	6E-169	Solyc01g009320.1	Solyc06g074140.1	0.2	0.85	0.23529412
**6**	201	1E-68	Solyc04g071030.1	Solyc11g005700.1	0	0	0
**18**	628	5E-67	Solyc11g008390.1	Solyc11g066040.1	0.35	3	0.11666667
**9**	321	5E-49	Solyc07g040940.2	Solyc12g008990.1	0.17	0.56	0.30357143
**8**	291	6E-163	Solyc06g062820.2	Solyc12g008990.1	0	0	0

Supplementary Table S5: The gene duplication analysis with Ka and Ks relation of 62 U-box E3 ubiquitin ligases.

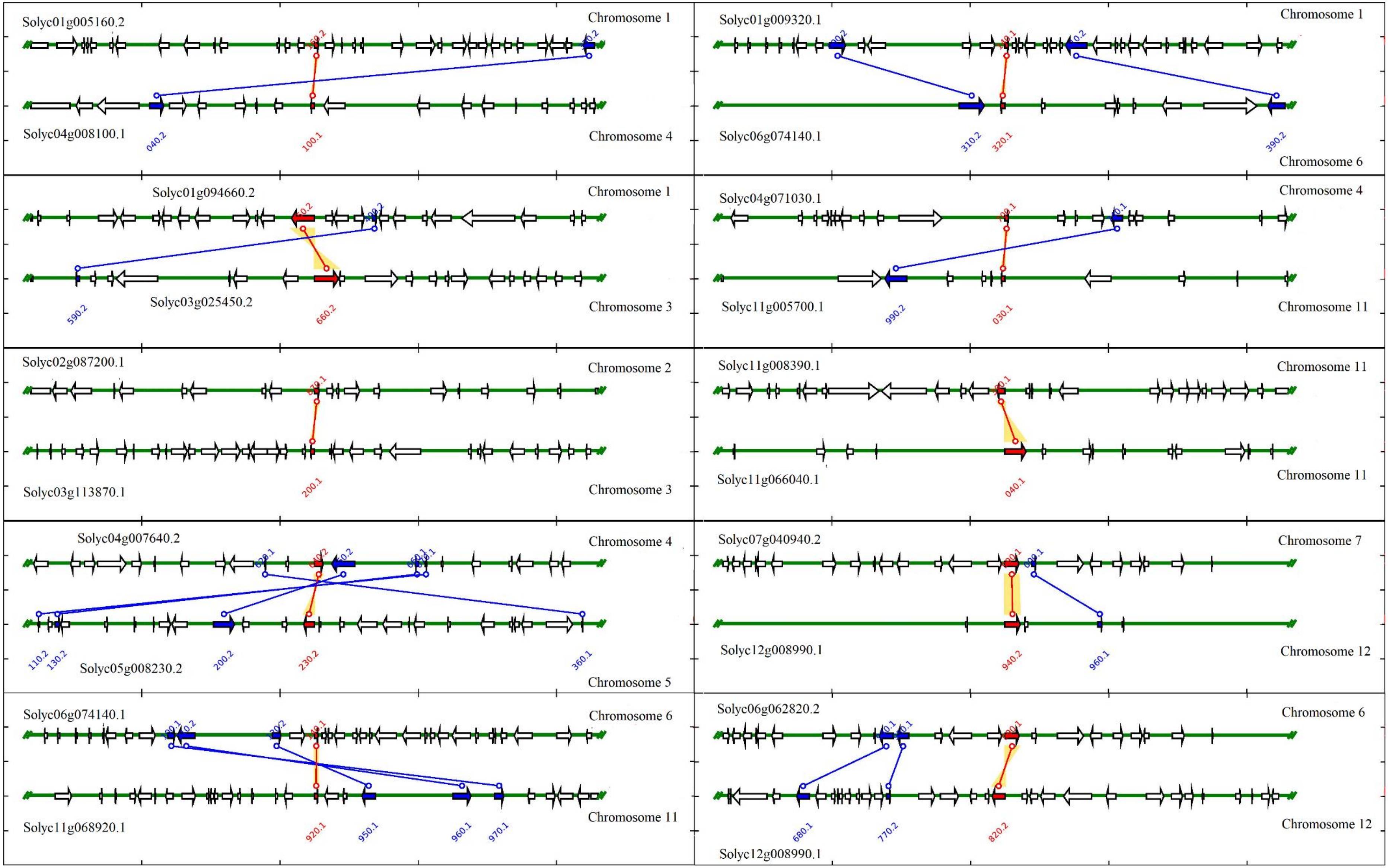

Supplementary Figure S6: Illustration of syntenic regions of the U-box E3 Ubiquitin Ligases in tomato. The U-box gene syntenic regions are marked with red arrows.

	Responsive Elements in various conditions																				
Gene	Heat	Cold	Auxin	Gibberelin	Abcisic Acid	Salysilic Acid	Jasmonic Acid	Fungal Elicitor	Defense	Ethylene	Endosperm	Cicadian	MYB binding in Drought	Alpha Amylase	Eliciter	Wound	Flavanoid	Seed specific	Zein Metabolism	Shoot specific and Light	Cell cycle	Root specific
SlU-box1	√						√	√	√	√												
SlU-box2	√					√			√		√	√										
SlU-box3		√		√				√			√	√	√		√	√						
SlU-box4	√				√				√	√	√	√		√			√					
SlU-box5						√					√	√						√				
SlU-box6	√					√				√	√	√			√							
SlU-box7						√	√				√	√			√							
SlU-box8	√		√						√		√				√							
SlU-box9						√					√	√										
SlU-box10	√								√		√	√	√		√							
SlU-box11	√			√	√	√	√				√	√			√				√			
SlU-box12	√			√		√																
SlU-box13	√		√	√	√	√	√		√	√	√	√	√		√					√		
SlU-box14	√					√			√													
SlU-box15	√			√	√		√	√		√	√											
SlU-box16				√		√			√		√											
SlU-box17	√			√	√		√		√		√						√					
SlU-box18	√	√	√			√		√	√		√	√							√			
SlU-box19	√	√		√	√	√			√		√	√	√		√							
SlU-box20							√		√	√	√	√	√			√						
SlU-box21	√										√		√							√		
SlU-box22	√			√		√	√				√								√			
SlU-box23							√		√		√	√			√							
SlU-box24	√			√	√		√	√		√		√	√						√	√	√	

SIU-box25	√					√			√	√		√	√									
SIU-box26	√								√			√	√									
SIU-box27				√		√	√	√			√	√										
SIU-box28	√	√	√	√		√			√		√	√										
SIU-box29	√			√					√	√	√	√	√									
SIU-box30				√	√	√	√	√	√		√		√									√
SIU-box31						√	√		√		√	√								√		√
SIU-box32	√	√		√		√			√		√						√					
SIU-box33	√		√						√		√											
SIU-box34	√				√		√		√		√											
SIU-box35	√							√	√	√		√							√			
SIU-box36	√			√	√	√			√	√	√	√							√			
SIU-box37		√		√							√								√			
SIU-box38								√	√	√	√											
SIU-box39	√		√		√				√	√	√											
SIU-box40									√		√	√							√			
SIU-box41	√			√		√			√		√		√									
SIU-box42	√	√	√	√	√	√		√	√		√	√					√					
SIU-box43									√	√	√	√	√									
SIU-box44	√		√						√				√									
SIU-box45	√	√	√			√	√		√		√		√			√						
SIU-box46					√	√			√	√	√	√	√									
SIU-box47					√	√		√	√			√										
SIU-box48		√		√			√			√	√	√	√									
SIU-box49			√			√	√			√	√	√	√						√			
SIU-box50	√			√							√	√										
SIU-box51	√			√	√	√				√	√	√	√			√						

SlU-box52				√	√	√	√	√	√		√					√						√
SlU-box53	√		√				√	√		√	√											
SlU-box54	√				√		√		√	√		√	√									
SlU-box55		√		√					√		√	√										
SlU-box56	√					√			√		√		√			√						
SlU-box57	√				√	√	√				√	√					√			√		
SlU-box58	√						√	√	√		√	√	√									
SlU-box59	√				√			√	√	√		√	√				√					
SlU-box60		√		√		√		√		√	√		√						√	√		
SlU-box61				√	√			√			√		√				√					
SlU-box62				√	√		√	√			√		√							√		

Supplementary Table S7: The occurrence of the various elements in the promoters of 62 tomato U-box E3 ubiquitin ligases.

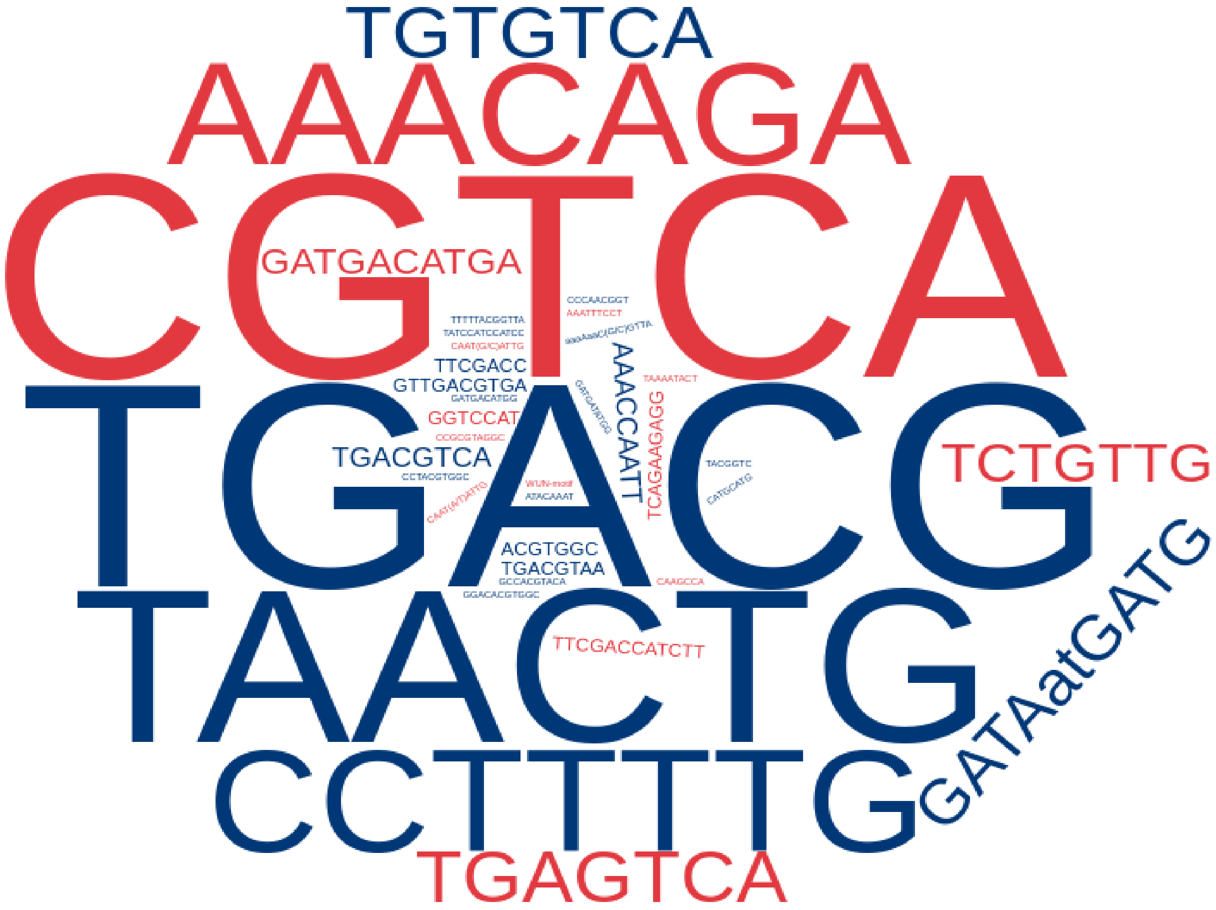

Supplementary Figure S8: The word cloud image of the promoter elements of 62 U-box E3 ubiquitin ligases in tomato. The size and intensity indicate the frequency and occurrence.

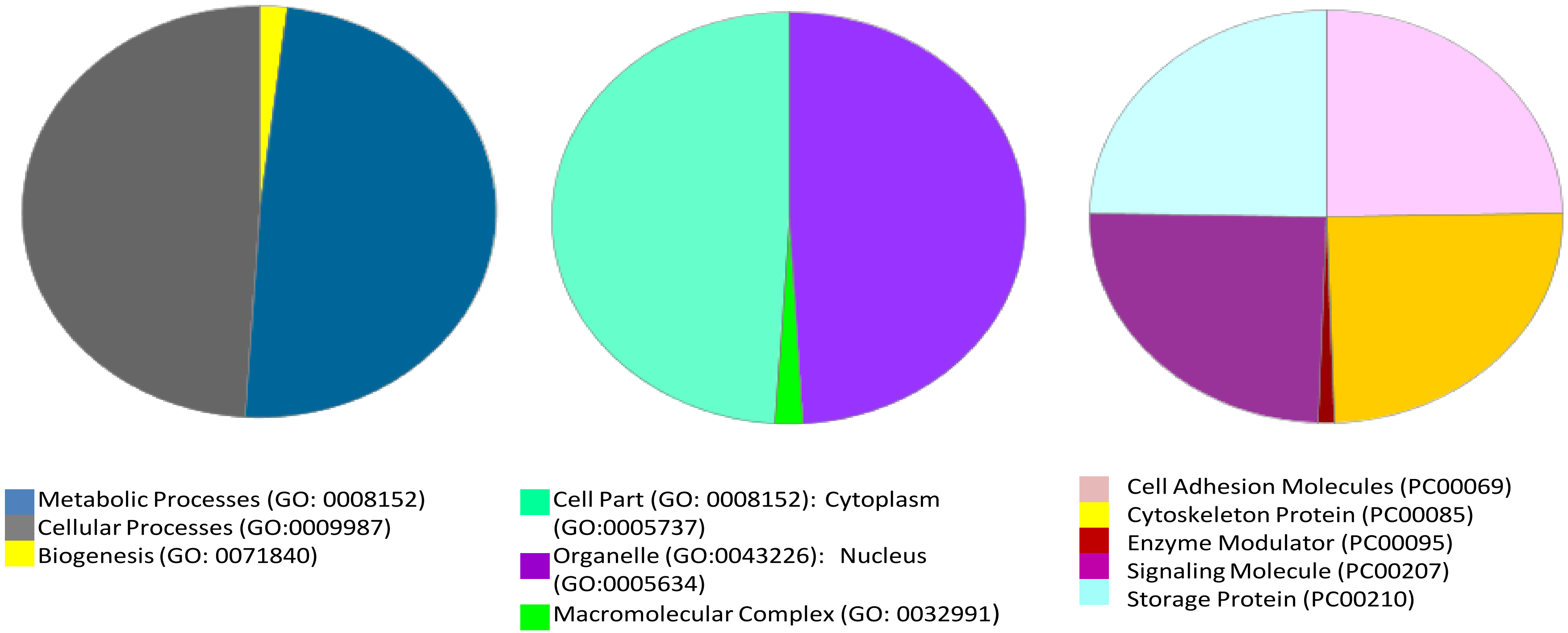

Supplementary Figure S9: The gene ontology analysis of the 62 U-box E3 ubiquitin ligases in tomato. The various predictions are shown in three pie charts.

Identifier	Species	Strain	Cultivar	Organ Type	Organ	Tissue	Stage
**C1**	S lyco	Wild Type	Micro.Tom	Vegetative	Root	Whole	nc
**C2**	S lyco	Wild Type	Heinz.1706	Vegetative	Root	Whole	NC.(root)
**C3**	S lyco	Wild Type	M82	Vegetative	Meristem	Shoot.Apical.Meristem	4.DAPL
**C4**	S lyco	Wild Type	M82	Vegetative	Meristem	Shoot.Apical.Meristem	5.DAPL
**C5**	S lyco	Wild Type	M82	Vegetative	Meristem	Whole	7DPG_EVM
**C6**	S lyco	Wild Type	M82	Vegetative	Meristem	Whole	10DPG_MVM
**C7**	S lyco	Wild Type	M82	Vegetative	Meristem	Shoot.Apical.Meristem	11DAPL
**C8**	S lyco	Wild Type	M82	Vegetative	Meristem	Whole	13DPG_LVM
**C9**	S lyco	Wild Type	M82	Vegetative	Meristem	Shoot.Apical.Meristem	14.DAPL
**C10**	S lyco	Wild Type	M82	Vegetative	Meristem	Whole	15DPG_TM
**C11**	S lyco	Wild Type	M82	Vegetative	Meristem	Whole	16DPG_FM
**C12**	S lyco	Wild Type	M82	Vegetative	Meristem	Whole	17DPG_SIM
**C13**	S lyco	Wild Type	M82	Vegetative	Meristem	Shoot.Apical.Meristem	17DAPL
**C14**	S lyco	Wild Type	M82	Vegetative	Meristem	Whole	19DPG_SYM
**C15**	S lyco	Wild Type	Rutgers	Vegetative	Meristem	Apical.Meristem	4weeks
**C16**	S lyco	Wild Type	Heinz.1706	Vegetative	Leaf	Whole	NC.(leaf)
**C17**	S lyco	Wild Type	Micro.Tom	Vegetative	Leaf	Whole	nc
**C18**	S lyco	Wild Type	M82	Vegetative	Leaf	Leaf.primordium	4DAPL
**C19**	S lyco	Wild Type	M82	Vegetative	Leaf	Leaf.primordium	5.DAPL
**C20**	S lyco	Wild Type	M82	Vegetative	Leaf	Leaf.primordium	11.DAPL
**C21**	S lyco	Wild Type	M82	Vegetative	Leaf	Leaf.primordium	14.DAPL
**C22**	S lyco	Wild Type	M82	Vegetative	Leaf	Leaf.primordium	17.DAPL

Supplementary Table S10: The different cultivars, tissues and conditions of tomato vegetative tissues used for the analysis of the RNA sequencing.

Identifier	Species	Strain	Cultivar	Organ Type	Organ	Tissue	Stage
**C1**	S pimp	Wild Type	LA.1589	Reproductive	Seed	Embryo	4.DPA
**C2**	S pimp	Wild Type	LA.1589	Reproductive	Seed	Endosperm	4.DPA
**C3**	S pimp	Wild Type	LA.1589	Reproductive	Seed	Seed.Coat	4.DPA
**C4**	S pimp	Wild Type	LA.1589	Reproductive	Seed	Funiculus	4.DPA
**C5**	S lyco	Wild Type	SUN1642	Reproductive	Seed	Whole	7.dpa
**C6**	S lyco	Wild Type	SUN1642	Reproductive	Seed	Whole	10.dpa
**C7**	S lyco	Wild Type	Micro.Tom	Reproductive	Seed	Whole	IMG_10DPA
**C8**	S lyco	Wild Type	Micro.Tom	Reproductive	Seed	Whole	MG_35DPA
**C9**	S lyco	Wild Type	Micro.Tom	Reproductive	Seed	Whole	Breaker_38DPA
**C10**	S lyco	Wild Type	Micro.Tom	Reproductive	Seed	Whole	Orange_41dpa
**C11**	S lyco	Wild Type	Micro.Tom	Reproductive	Seed	Whole	Red_44DPA
**C12**	S lyco	Wild Type	M82	Reproductive	Flower	Meristem	8DPG_EVM
**C13**	S lyco	Wild Type	Micro.Tom	Reproductive	Flower	Petal	nc
**C14**	S lyco	Wild Type	Heinz.1706	Reproductive	Flower	Whole	NC.(flower.bud)
**C15**	S lyco	Wild Type	Heinz.1706	Reproductive	Flower	Whole	NC.(flower)
**C16**	S lyco	Wild Type	Micro.Tom	Reproductive	Flower	Whole	Bud
**C17**	S lyco	Wild Type	Micro.Tom	Reproductive	Flower	Whole	Bud.3.mm
**C18**	S lyco	Wild Type	Micro.Tom	Reproductive	Flower	Whole	Anthesis
**C19**	S pimp	Wild Type	LA.1589	Reproductive	Fruit	Ovule	Anthesis
**C20**	S pimp	Wild Type	LA.1589	Reproductive	Fruit	Pericarp	Anthesis
**C21**	S pimp	Wild Type	LA.1589	Reproductive	Fruit	Placenta	Anthesis
**C22**	S pimp	Wild Type	LA.1589	Reproductive	Fruit	Septum	Anthesis

**C23**	S lyco	Wild Type	Moneymaker	Reproductive	Fruit	Ovule	0DPAe
**C24**	S lyco	Wild Type	Moneymaker	Reproductive	Fruit	Ovule.wall/pericarp	0DPAe
**C25**	S lyco	Wild Type	Moneymaker	Reproductive	Fruit	Ovule	1DPA
**C26**	S lyco	Wild Type	Moneymaker	Reproductive	Fruit	Ovule.wall/pericarp	1DPA
**C27**	S lyco	Wild Type	Moneymaker	Reproductive	Fruit	Ovule	2DPA
**C28**	S lyco	Wild Type	Moneymaker	Reproductive	Fruit	Ovule.wall/pericarp	2DPA
**C29**	S lyco	Wild Type	Moneymaker	Reproductive	Fruit	Ovule	5DPA
**C30**	S lyco	Wild Type	Moneymaker	Reproductive	Fruit	Ovule.wall/pericarp	5DPA
**C31**	S pimp	Wild Type	LA.1589	Reproductive	Fruit	Pericarp	4.DPA
**C32**	S pimp	Wild Type	LA.1589	Reproductive	Fruit	Placenta	4.DPA
**C33**	S pimp	Wild Type	LA.1589	Reproductive	Fruit	Septum	4.DPA
**C34**	S lyco	Wild Type	SUN1642	Reproductive	Fruit	Septum_Seed	4.dpa
**C35**	S lyco	Wild Type	SUN1642	Reproductive	Fruit	Pericarp_Exocarp	4.dpa
**C36**	S lyco	Wild Type	Micro.Tom	Reproductive	Fruit	Whole	4.dpa
**C37**	S lyco	Wild Type	SUN1642	Reproductive	Fruit	Septum	7.dpa
**C38**	S lyco	Wild Type	SUN1642	Reproductive	Fruit	Pericarp	7.dpa
**C39**	S lyco	Wild Type	SUN1642	Reproductive	Fruit	Septum	10.dpa
**C40**	S lyco	Wild Type	SUN1642	Reproductive	Fruit	Pericarp	10.dpa
**C41**	S lyco	Wild Type	Ailsa.Craig	Reproductive	Fruit	Top_section	Immature.Green.10.dpa
**C42**	S lyco	Wild Type	Ailsa.Craig	Reproductive	Fruit	Middle_section	Immature.Green.10.dpa
**C43**	S lyco	Wild Type	Ailsa.Craig	Reproductive	Fruit	Bottom_section	Immature.Green.10.dpa
**C44**	S lyco	Wild Type	Micro.Tom	Reproductive	Fruit	Flesh	IMG_10DPA
**C45**	S lyco	Wild Type	Micro.Tom	Reproductive	Fruit	Peel	IMG_10DPA

**C46**	S lyco	Wild Type	Ailsa.Craig	Reproductive	Fruit	Pericarp	Immature.Green.14.dpa
**C47**	S lyco	Wild Type	M82	Reproductive	Fruit	Whole	Immature.Green.15.dpa
**C48**	S lyco	Wild Type	Ailsa.Craig	Reproductive	Fruit	Whole	Immature.Green.17.dpa
**C49**	S lyco	Wild Type	Ailsa.Craig	Reproductive	Fruit	Top_section	Immature.Green.20.dpa
**C50**	S lyco	Wild Type	Ailsa.Craig	Reproductive	Fruit	Middle_section	Immature.Green.20.dpa
**C51**	S lyco	Wild Type	Ailsa.Craig	Reproductive	Fruit	Bottom_section	Immature.Green.20.dpa
**C52**	S lyco	Wild Type	Heinz.1706	Reproductive	Fruit	Whole	1.cm
**C53**	S lyco	Wild Type	Heinz.1706	Reproductive	Fruit	Whole	2.cm
**C54**	S lyco	Wild Type	Heinz.1706	Reproductive	Fruit	Whole	3.cm
**C55**	S lyco	Wild Type	Ailsa.Craig	Reproductive	Fruit	Top_section	Mature.Green
**C56**	S lyco	Wild Type	Ailsa.Craig	Reproductive	Fruit	Middle_section	Mature.Green
**C57**	S lyco	Wild Type	Ailsa.Craig	Reproductive	Fruit	Bottom_section	Mature.Green
**C58**	S lyco	Wild Type	Micro.Tom	Reproductive	Fruit	Flesh	MG_35DPA
**C59**	S lyco	Wild Type	Micro.Tom	Reproductive	Fruit	Peel	MG_35DPA
**C60**	S lyco	Wild Type	Heinz.1706	Reproductive	Fruit	Whole	Mature.Green
**C61**	S lyco	Wild Type	Ailsa.Craig	Reproductive	Fruit	Whole	Mature.green_35DPA
**C62**	S lyco	Wild Type	Micro.Tom	Reproductive	Fruit	Flesh	Breaker_38DPA
**C63**	S lyco	Wild Type	Micro.Tom	Reproductive	Fruit	Peel	Breaker_38DPA
**C64**	S lyco	Wild Type	Ailsa.Craig	Reproductive	Fruit	Whole	Mature.Green.39.dpa
**C65**	S lyco	Wild Type	Micro.Tom	Reproductive	Fruit	Flesh	Orange_41dpa
**C66**	S lyco	Wild Type	Micro.Tom	Reproductive	Fruit	Peel	Orange_41dpa
**C67**	S lyco	Wild Type	Ailsa.Craig	Reproductive	Fruit	Whole	Breaker.42.dpa
**C68**	S lyco	Wild Type	Ailsa.Craig	Reproductive	Fruit	Top_section	Breaker

**C69**	S lyco	Wild Type	Ailsa.Craig	Reproductive	Fruit	Middle_section	Breaker
**C70**	S lyco	Wild Type	Ailsa.Craig	Reproductive	Fruit	Bottom_section	Breaker
**C71**	S lyco	Wild Type	Heinz.1706	Reproductive	Fruit	Whole	Breaker
**C72**	S lyco	Wild Type	Micro.Tom	Reproductive	Fruit	Flesh	Red_44DPA
**C73**	S lyco	Wild Type	Micro.Tom	Reproductive	Fruit	Peel	Red_44DPA
**C74**	S lyco	Wild Type	Ailsa.Craig	Reproductive	Fruit	Whole	Pink_45DPA
**C75**	S lyco	Wild Type	Ailsa.Craig	Reproductive	Fruit	Top_section	Breaker.+.5
**C76**	S lyco	Wild Type	Ailsa.Craig	Reproductive	Fruit	Middle_section	Breaker.+.5
**C77**	S lyco	Wild Type	Ailsa.Craig	Reproductive	Fruit	Bottom_section	Breaker.+.5
**C78**	S lyco	Wild Type	Ailsa.Craig	Reproductive	Fruit	Whole	Breaker.+.(7_10)
**C79**	S lyco	Wild Type	M82	Reproductive	Fruit	Whole	Breaker.+.10
**C80**	S lyco	Wild Type	Ailsa.Craig	Reproductive	Fruit	Top_section	Breaker.+.10
**C81**	S lyco	Wild Type	Ailsa.Craig	Reproductive	Fruit	Middle_section	Breaker.+.10
**C82**	S lyco	Wild Type	Ailsa.Craig	Reproductive	Fruit	Bottom_section	Breaker.+.10
**C83**	S lyco	Wild Type	Heinz.1706	Reproductive	Fruit	Whole	Breaker.10
**C84**	S lyco	Wild Type	Ailsa.Craig	Reproductive	Fruit	Whole	Fully.ripe.52.dpa

Supplementary Table S11: The different cultivars, tissues and conditions of tomato reproductive tissues used for the analysis of the RNA sequencing.

Identifier	Species	Strain	Cultivar	Organ Type	Organ	Tissue	Stage	Treatment	Duration	Pathogen
**C1**	S lyco	Wild Type	M82	Vegetative	Seedling	Whole	10.dpg	sun	-	-
**C2**	S lyco	Wild Type	M82	Vegetative	Seedling	Whole	10.dpg	shade	-	-
**C3**	S lyco	Wild Type	M82	Vegetative	Seedling	Whole	10.dpg	shade	-	-
**C4**	S lyco	Wild Type	M82	Vegetative	Seedling	Leaf	2.young.leaves	PAC	3d	-
**C5**	S lyco	Wild Type	M82	Vegetative	Seedling	Leaf	2.young.leaves	PAC_GA	3d_30min	-
**C6**	S lyco	Wild Type	M82	Vegetative	Root	Whole	10.dpg	sun	-	-
**C7**	S lyco	Wild Type	M82	Vegetative	Root	Whole	10.dpg	shade	-	-
**C8**	S lyco	Wild Type	Micro.Tom	Vegetative	Root	Whole	14.dpg	DMSO	24h	-
**C9**	S lyco	Wild Type	Micro.Tom	Vegetative	Root	Whole	14.dpg	Cytok	24h	-
**C10**	S lyco	Wild Type	Micro.Tom	Vegetative	Root	Whole	14.dpg	Auxin	24h	-
**C11**	S lyco	Wild Type	Micro.Tom	Vegetative	Root	Lateral	14.dpg	DMSO	24h	-
**C12**	S lyco	Wild Type	Micro.Tom	Vegetative	Root	Lateral	14.dpg	Cytok	24h	-
**C13**	S lyco	Wild Type	Micro.Tom	Vegetative	Root	Lateral	14.dpg	Auxin	24h	-
**C14**	S lyco	Wild Type	Micro.Tom	Vegetative	Root	Tip	14.dpg	DMSO	24h	-
**C15**	S lyco	Wild Type	Micro.Tom	Vegetative	Root	Tip	14.dpg	Cytok	24h	-
**C16**	S lyco	Wild Type	Micro.Tom	Vegetative	Root	Tip	14.dpg	Auxin	24h	-
**C17**	S lyco	Wild Type	M82	Vegetative	Meristem	Shoot.Apical.Meristem	19.DAPL	Shade	Cont	-
**C18**	S lyco	Wild Type	M82	Vegetative	Meristem	Shoot.Apical.Meristem	19.DAPL	Sun	Cont	-
**C19**	S lyco	Wild Type	M82	Vegetative	Meristem	Shoot.Apical.Meristem	19.DAPL	Tr Shade	28 h	-
**C20**	S lyco	Wild Type	M82	Vegetative	Meristem	Shoot.Apical.Meristem	19.DAPL	CTRL Sun Tr Shade	Cont	-
**C21**	S lyco	Wild Type	M82	Vegetative	Meristem	Whole	35.dpg	sun	-	-

**C22**	S lyco	Wild Type	M82	Vegetative	Meristem	Whole	35.dpg	shade	-	-
**C23**	S lyco	Wild Type	M82	Vegetative	Stem	Whole	50.dpg	shade	-	-
**C24**	S lyco	Wild Type	M82	Vegetative	Stem	Whole	50.dpg	sun	-	-
**C25**	S lyco	Wild Type	M82	Vegetative	Leaf	Leaf.primordium	19.DAPL	Shade	Cont	-
**C26**	S lyco	Wild Type	M82	Vegetative	Leaf	Leaf.primordium	19.DAPL	Sun	Cont	-
**C27**	S lyco	Wild Type	M82	Vegetative	Leaf	Leaf.primordium	19.DAPL	Tr Shade	28 h	-
**C28**	S lyco	Wild Type	M82	Vegetative	Leaf	Leaf.primordium	19.DAPL	Sun Tr shift	Cont	-
**C29**	S lyco	Wild Type	Micro.Tom	Vegetative	Leaf	Whole	13.dpg	DMSO	2h	-
**C30**	S lyco	Wild Type	Micro.Tom	Vegetative	Leaf	Whole	13.dpg	Cytok	2h	-
**C31**	S lyco	Wild Type	Micro.Tom	Vegetative	Leaf	Whole	13.dpg	DMSO	24h	-
**C32**	S lyco	Wild Type	Micro.Tom	Vegetative	Leaf	Whole	13.dpg	Cytok	24h	-
**C33**	S lyco	Wild Type	Micro.Tom	Vegetative	Leaf	Whole	35.dpg	DMSO	2h	-
**C34**	S lyco	Wild Type	Micro.Tom	Vegetative	Leaf	Whole	35.dpg	Cytok	2h	-
**C35**	S lyco	Wild Type	Micro.Tom	Vegetative	Leaf	Whole	35.dpg	DMSO	24h	-
**C36**	S lyco	Wild Type	Micro.Tom	Vegetative	Leaf	Whole	35.dpg	Cytok	24h	-
**C37**	S lyco	Wild Type	Hongtaiyang.903	Vegetative	Leaf	Whole	46.dpg	mock	0h	-
**C38**	S lyco	Wild Type	Hongtaiyang.903	Vegetative	Leaf	Whole	46.dpg	mock	24h	-
**C39**	S lyco	Wild Type	Hongtaiyang.903	Vegetative	Leaf	Whole	46.dpg	ABA	24h	-
**C40**	S lyco	Wild Type	Hongtaiyang.903	Vegetative	Leaf	Whole	46.dpg	mock	48h	-
**C41**	S lyco	Wild Type	Hongtaiyang.903	Vegetative	Leaf	Whole	46.dpg	ABA	48h	-
**C42**	S lyco	Wild Type	M82	Vegetative	Leaf	Whole	50.dpg	sun	-	-
**C43**	S lyco	Wild Type	M82	Vegetative	Leaf	Whole	50.dpg	shade	-	-

**C44**	S lyco	Wild Type	Moneymaker	Vegetative	Leaf	Whole	56.DAPL	Ctrl	-	-
**C45**	S lyco	Wild Type	Moneymaker	Vegetative	Leaf	Whole	56.DAPL	HS_2	1h_39°C	-
**C46**	S lyco	Wild Type	Hazera.3042	Reproductive	Flower	Pollen	Anthesis	Ctrl	Cont	-
**C47**	S lyco	Wild Type	Hazera.3042	Reproductive	Flower	Pollen	Anthesis	HS_1	Cont	-
**C48**	S lyco	Wild Type	M82	Reproductive	Flower	Whole	50.dpg	sun	-	-
**C49**	S lyco	Wild Type	M82	Reproductive	Flower	Whole	50.dpg	shade	-	-
**C50**	S lyco	Wild Type	Moneymaker	Reproductive	Flower	Anthers	56.DAPL	Ctrl	-	-
**C51**	S lyco	Wild Type	Moneymaker	Reproductive	Flower	Anthers	56.DAPL	HS_2	1h_39°C	-
**C52**	S lyco	Wild Type	M82	Reproductive	Fruit	Whole	IG+MG	sun	-	-
**C53**	S lyco	Wild Type	M82	Reproductive	Fruit	Whole	IG+MG	shade	-	-
**C54**	S lyco	Wild Type	Micro.Tom	Reproductive	Fruit	Whole	Mature.Green	Ctrl	0h	-
**C55**	S lyco	Wild Type	Micro.Tom	Reproductive	Fruit	Whole	Mature.Green	Ctrl	48h	-
**C56**	S lyco	Wild Type	Micro.Tom	Reproductive	Fruit	Whole	Mature.Green	IAA	48h	-
**C57**	S lyco	Wild Type	Micro.Tom	Reproductive	Fruit	Whole	Mature.Green	ACC	48h	-
**C58**	S lyco	Wild Type	Micro.Tom	Reproductive	Fruit	Whole	Mature.Green	IAA+ACC	48h	-

Supplementary Table S12: The different cultivars and tissues treated with abiotic stress and various hormones for the analysis of the RNA sequencing.

Identifier	Species	Strain	Cultivar	Organ Type	Organ	Tissue	Stage	Treatment	Duration	Pathogen
**C1**	S lyco	Wild Type	Avigail.(870)	Vegetative	Root	Whole	15.dpg._0dpi	Meljav	0d	Meloidogyne.javanica
**C2**	S lyco	Wild Type	Avigail.(870)	Vegetative	Root	Whole	15.dpg._2dpi	Meljav	2d	Meloidogyne.javanica
**C3**	S lyco	Wild Type	Avigail.(870)	Vegetative	Root	Whole	15.dpg._5dpi	Meljav	5d	Meloidogyne.javanica
**C4**	S lyco	Wild Type	Avigail.(870)	Vegetative	Root	Whole	15.dpg._15dpi	Meljav	15d	Meloidogyne.javanica
**C5**	S lyco	Wild Type	Ailsa.Craig	Vegetative	Leaf	Whole	nc	VIGS_AGO1	3h	Agrobacterium.tumefaciens.GV3101
**C6**	S lyco	Wild Type	Ailsa.Craig	Vegetative	Leaf	Whole	nc	VIGS_Ctrl	3h	Agrobacterium.tumefaciens.GV3101
**C7**	S lyco	Wild Type	TYLCV-resistant.CLN2777A	Vegetative	Leaf	Whole	2.leaves	TYLC	0d	Tomato.yellow.leaf.curl.virus
**C8**	S lyco	Wild Type	TYLCV-resistant.CLN2777A	Vegetative	Leaf	Whole	2.leaves	TYLC	3-7d	Tomato.yellow.leaf.curl.virus
**C9**	S lyco	Wild Type	TYLCV-susceptible.TMXA48-4-0	Vegetative	Leaf	Whole	2.leaves	TYLC	0d	Tomato.yellow.leaf.curl.virus
**C10**	S lyco	Wild Type	TYLCV-susceptible.TMXA48-4-0	Vegetative	Leaf	Whole	2.leaves	TYLC	3-7d	Tomato.yellow.leaf.curl.virus
**C11**	S lyco	Wild Type	Moneymaker	Reproductive	Fruit	Whole	Breaker	Ctrl	-	-
**C12**	S lyco	Wild Type	Moneymaker	Reproductive	Fruit	Whole	Breaker	Fu_mos	-	Funneliformis.mosseae

Supplementary Table S13: The different cultivars and tissues treated with various pathogens (biotic stress) for the analysis of the RNA sequencing.
